# Development and utility of SSR markers based on *Brassica* sp. whole-genome in triangle of U

**DOI:** 10.3389/fpls.2023.1259736

**Published:** 2024-01-08

**Authors:** Nairan Sun, Jisuan Chen, Yuqi Wang, Iqbal Hussain, Na Lei, Xinyan Ma, Weiqiang Li, Kaiwen Liu, Hongrui Yu, Kun Zhao, Tong Zhao, Yi Zhang, Xiaolin Yu

**Affiliations:** ^1^ Group of Vegetable Breeding, Hainan Institute of Zhejiang University, Sanya, China; ^2^ Department of Horticulture, College of Agriculture and Biotechnology, Zhejiang University, Hangzhou, China; ^3^ Zhejiang Provincial Key Laboratory of Horticultural Plant Integrative Biology, Hangzhou, China; ^4^ Department of Supply Chain, Ningbo Haitong Food Technology Co., Ltd., Ningbo, China; ^5^ Section of Horticulture and Landscape Architecture, Harbin Academy of Agricultural Sciences, Harbin, China

**Keywords:** *Brassica* L, simple sequence repeats, microsatellite, primer development, genomewide, B-genome specific SSR marker

## Abstract

**Introduction:**

Simple sequence repeats (SSR), also known as microsatellites, are crucial molecular markers in both animals and plants. Despite extensive previous research on SSRs, the development of microsatellite markers in Brassica crops remains limited and inefficient.

**Methods:**

Krait software was used to identify microsatellites by genome-wide and marker development based on three recently sequenced basic species of *Brassica* crops in the triangle of U (*Brassica* rapa, *B. nigra* and *B. oleracea*), as well as three allotetraploids (*B. juncea, B. napus* and *B. carinata*) using public databases. Subsequently, the primers and the characteristics of microsatellites for most of them were accordingly designed on each chromosome of each of the six Brassica species, and their physical locations were identified,and the cross-transferability of primers have been carried out. In addition, a B-genome specific SSR marker was screened out.

**Results:**

A total of 79341, 92089, 125443, 173964, 173604, and 222160 SSR loci have been identified from the whole genome sequences of *Brassica* crops within the triangle of U crops, *B. rapa* (AA), *B. nigra* (BB), *B. oleracea* (CC), *B. napus* (AACC), *B. juncea* (AABB) and *B. carinata* (BBCC), respectively. Comparing the number distribution of the three allotetraploid SSR loci in the three subgenomes AA, BB and CC, results indicate that the allotetraploid species have significant reduction in the number of SSR loci in the genome compared with their basic diploid counterparts. Moreover, we compared the basic species with their corresponding varieties, and found that the microsatellite characters between the allotetraploids and their corresponding basic species were very similar or almost identical. Subsequently, each of the 40 SSR primers was employed to investigate the polymorphism potential of *B. rapa* (85.27%), *B. nigra* (81.33%) and *B. oleracea* (73.45%), and *B. rapa* was found to have a higher cross-transfer rate among the basic species in the triangle of U. Meanwhile, a B-genome specific SSR marker, *BniSSR23228* possessing the (AAGGA)_3_ sequence characteristics was obtained, and it located in chromosome B3 with a total length of 97 bp.

**Discussion:**

In this study, results suggest that the pattern of distribution may be highly conserved during the differentiation of basic *Brassica* species and their allotetraploid counterparts. Our data indicated that the allotetraploidization process resulted in a significant reduction in SSR loci in the three subgenomes AA, BB and CC. The reasons may be partial gene dominated chromosomal homologous recombination and rearrangement during the evolution of basic diploid species into allotetraploids. This study provides a basis for future genomics and genetic research on the relatedness of *Brassica* species.

## 1 Introduction

**Figure 1:**
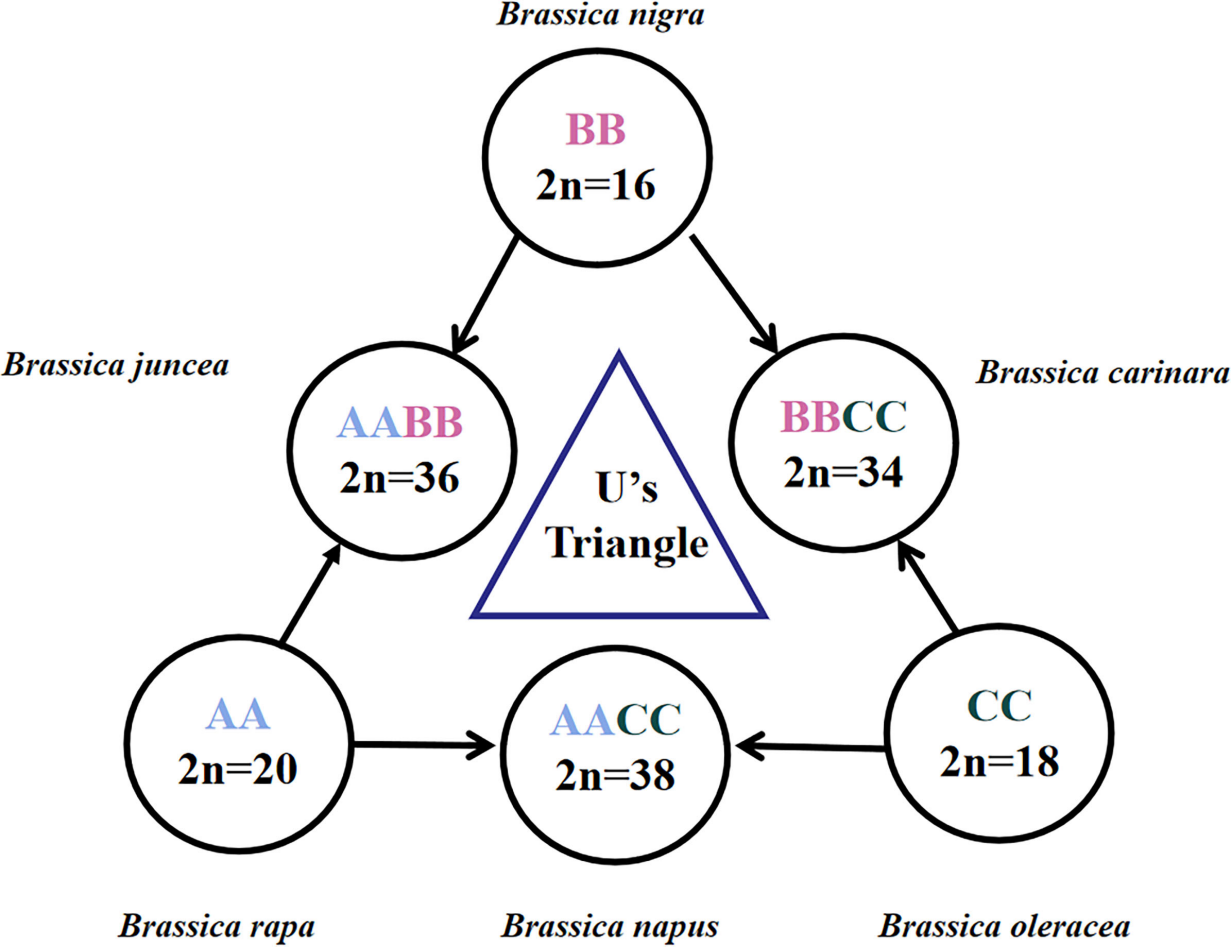
*Brassica* species in the triangle of U. The three diploid basic species are referred to by AA, BB and CC genomes, and the three allotetraploid species are referred to by AABB, AACC and BBCC. The diploid chromosome number (2n) is shown. The image is adapted from U (1935).

*Brassica*, as a diverse and important genus within the cruciferous family, includes many important vegetable and oilseed crops for human consumption or food production, such as Chinese cabbage, turnip, cabbage, cauliflower, broccoli, Brussels sprouts, kohlrabi, kale, collards, mustard, and rapeseed. These crops can be stored for a long time and provide sufficient food reserves in winter. Not only several *Brassica* species are economically important oil seeds, spices and vegetables, but also they are rich in essential nutrients such as vitamin C and glucosinolates, which has been associated with a reduced risk of many cancers (Kristal and Lampe, 2002). The genetic relationships between the top six *Brassica* species can be described by the triangle of U model (Nagaharu, 1935) (**Figure 1**). Therein, three ancestral diploid species *B. rapa* (A genome, n=10), *B. nigra* (B genome, n=8) and *B. oleracea* (C genome. n=9) have been cross-bred over time to produce three allotetraploids: *B. juncea* (AB genome, n=18), *B. napus* (AC genome, n=19) and *B. carinata* (BC genome, n=17). Some *Brassica* crops were also found to be capable in crossing with other important cruciferous crops such as wild radish (*Raphanus*) (Beckie et al., 2003; FitzJohn et al., 2007). This potential to hybridize with a wide range of inbreds and the diversity of non-domesticated forms of key crop species makes *Brassica* an integral part of global gene banks.

The detection of DNA sequence variation is a crucial step in studying the *Brassica* genome. Over the past two decades, various molecular markers have been used in genetic breeding studies of *Brassica*, such as restriction fragment length polymorphisms (RFLP), random amplified polymorphic DNA (RAPD), amplified fragment length polymorphisms (AFLP), simple sequence repeats (SSRs), sequence-related amplified polymorphisms (SRAP), sequence-characterized amplified regions (SCAR), and single nucleotide polymorphisms (SNP) (Ananga et al., 2006; Rahman et al., 2010; Zeng et al., 2010; Christensen et al., 2011; Panigrahi et al., 2011; Rezaeizad et al., 2011; Shirasawa et al., 2011). Among these molecular markers, SSRs or microsatellites are characterized by high polymorphism, reproducibility, ease of detection by polymerase chain reaction (PCR), co-dominance, adaptability, transferability, and genomic abundance. Thus, SSRs have been widely used in genetic diversity studies, quantitative trait loci and genetic mapping analysis, gene localization, germplasm classification and evolution and comparative genomics, and it is still one of the important molecular markers in genetic breeding research (Wang et al., 2014).

Traditional methods for developing SSRs involve the probe hybridization of genomic and cDNA libraries containing repetitive motifs, followed by DNA sequencing (Lowe et al., 2004), or the *in silico* analysis of publicly available bacterial artificial chromosome (BAC) sequences (Burgess et al., 2006; Xu et al., 2010), genomic survey sequences and whole-genome shotgun sequences (Cheng et al., 2009; Li et al., 2011). These procedures are time-consuming, costly and labor-intensive; however, with the expansion of DNA sequence information in public databases, the development of SSRs from publicly available DNA sequences has become a rapid and cost-effective alternative (McCouch et al., 2002; Song et al., 2005; Shoemaker et al., 2008). Currently, genome-wide SSR-based development is commonly used in crops such as cocoa, grapes, maize, kidney beans, and prunes (Cai et al., 2009; Cao et al., 2013; Qu and Liu, 2013). This approach has also proved useful in developing SSRs in expressed sequence tags in many agricultural crops, including rice, wheat, cotton, barley, groundnut, cowpea, and radish (Cardle et al., 2000; Kantety et al., 2002; La Rota et al., 2005; Park et al., 2005; Liang et al., 2009; Gupta and Gopalakrishna, 2010; Shirasawa et al., 2011).

With the rapid advancement of whole genome sequencing technology, the genome sequence of cabbage has been released and is available online (http://www.ocri-genomics.org/bolbase/index.html) (Liu et al., 2014). Genome sequences provide a powerful pool of information for genome-wide microsatellite characterization. At the same time, studies on the development of SSRs based on the whole genome of *Brassica* have been limited (Shi et al., 2014). Therefore, in this study, we analyzed the genome-wide SSR information distribution of six *Brassica* species, and located the physical position of SSRs on each chromosome to analyze the relatedness between these species. To evaluate the newly developed genome-wide SSR markers in representative self-crossed lines, we attached these SSR primers of these species as **Supplementary Files** and screened a number of specific SSR markers by PCR amplification. Moreover, a B-genome specific SSR marker, *BniSSR23228*, was screened out. These results provide great value in relevant research fields including introgression line tracking, genetic diversity analysis, marker-assisted breeding, and so on.

## 2 Materials and methods

### 2.1 Source of the whole genome sequence

The genome sequences of three basic species [*B. rapa* (Brara_Chiifu_V3.5), *B. nigra* (Brana_ NI100_V2) and *B. oleracea* (Braol_JZS_V2.0)] and three allotetraploids [*B. juncea* (Braju_tum_V1.5), *B. napus* (Brana_Dar_V5) and *B. carinata* (http://brassicadb.bio2db.com/download.html)] of the genus *Brassica* were downloaded from the *Brassica* Info (http://www.Brassica.info/) website (Chen et al., 2010; Liu et al., 2014). The sequences obtained for *B. rapa, B. nigra, B. oleracea, B. juncea, B. napus* and *B. carinata* were 353140194 bp, 506000232bp, 561157886 bp, 937030072 bp, 850292103 bp, and 1086987601 bp in length, respectively.

### 2.2 SSR screening

The Krait identification tool was employed to search for the presence of SSR motifs in the genomic sequences (Du et al., 2018). The parameters were set as follows: the minimum number of repeat units was 12 single nucleotides, 7 dinucleotides, 5 trinucleotides, 4 tetranucleotides and pentanucleotides, and 4 hexanucleotides. The frequency and length of the searched SSRs were counted and analyzed.

### 2.3 Genomic SSR primer design

The primer pairs on both sides of the SSR loci were designed using Krait software (Du et al., 2018). The main parameters were set as follows: the primer length was controlled between 18 and 27 bp, with an optimal size of 20 bp; the melting temperature was 58°C to 65°C, with an optimal temperature of 60°C; the GC content was in the range of 30% to 80%; and the predicted PCR product was in the range of 100-300 bp. All other parameters were set as default.

### 2.4 Plant material and DNA extraction

The plant materials used in the experiment were from *B. rapa* (AA), *B. nigra* (BB), *B. oleracea* (CC), *B. juncea* (AABB), *B. napus* (AACC), *B. carinata* (BBCC), *Raphanus sativus* (RR) and *Arabidopsis thaliana* (At) specimens. The total genomic DNA was extracted from young frozen leaf tissues using the SDS method. The genomic DNA concentrations (ng/µL) were adjusted to an experimentally specific 100 ng/µL using a NanoDrop 1000 spectrophotometer (Thermo Fisher Scientific, USA).

### 2.5 Detection of the transferability of SSR markers

From the SSR primers designed based on the genomes of the six chosen species, 40 pairs of primers were randomly selected in each of the three diploid species, *B. rapa*, *B. nigra* and *B. oleracea*. To perform PCR amplification, a reaction mixture containing 1 µL (100 ng/µL) of template genomic DNA, 1µL (10 µmol/L) of each primer and 12.5 µL of 2×T5 Super PCR Mix (PAGE) buffer was added, followed by the addition of ddH_2_O to 25 µL total volume of the reaction mixture. The PCR assay amplification procedure included pre-denaturation at 94°C for 3 min, then 35 cycles involving denaturation at 94°C for 30 s, annealing at 53°C for 30 s, and extension at 72°C for 30 s, and finally, extension at 72°C for 7 min. The PCR reaction procedure was performed on a BIO-RAD S1000TM Thermal Cycler instrument, and samples were stored at 4°C. The SSR cross-transfer rate refers to the number of the amplified bands obtained from the other seven related species except for itself/total bands×100%.

### 2.6 Polyacryamide gel electrophoresis detection of the PCR product

The PCR product was detected by 12% PAGE following a method modified from “Molecular Cloning: A Laboratory Manual” (Sambrook et al., 2001).

### 2.7 Statistical analysis

The obtained SSR marker loci were analyzed and calculated using MG2C (http://mg2c.iask.in/mg2c_v2.1/) to locate the physical position of the SSR on each chromosome (Chao et al., 2021). A binary matrix of ‘1’ and ‘0’ was prepared for the SSR marker allele data for all genotypes. The polymorphic information content (PIC) values, gene diversity and heterozygosity were calculated using PowerMarker 3.0 software (Liu and Muse, 2005).

## 3 Results

### 3.1 Genome-wide SSR identification of *Brassica* species in the triangle of U

From the genomic sequences of *B. rapa*, *B. nigra*, *B. oleracea*, *B. napus*, *B. juncea*, and *B. carinata* with lengths of 340, 490, 541, 893, 824 and 1085.44 Mb, respectively, we identified 79341, 92089, 125443, 173964, 173604 and 222160 complete mono-nucleotide to hexanucleotide repeat sequence microsatellites with total frequencies of 226, 182, 223.6, 231.31, 235.12 and 204.42 loci per Mb, respectively (**Tables 1**, **2**).

**Table 1:** Distribution of the main SSR types in the genomes of the three basic species.

Motif	*B*. *rapa*		*B*. *oleracea*		*B*. *nigra*	
	Number (%)	Total Length (%)	Number (%)	Total Length (%)	Number (%)	Total Length (%)
Mono	34314 (43.25)	507875 (34.34)	62204 (49.59)	953125 (42.83)	31373 (34.07)	410790 (20.93)
A	32275 (40.68)	470914 (31.84)	57629 (45.94)	862833 (38.78)	31308 (34.00)	409999 (20.89)
C	2039 (2.57)	36961 (2.50)	4575 (3.65)	90292 (4.06)	65 (0.07)	791 (0.04)
Di	28637 (36.09)	663746 (44.88)	41560 (33.13)	892714 (40.12)	38893 (42.23)	1037216 (52.84)
AT	18181 (22.92)	403818 (27.30)	27267 (21.74)	577378 (25.95)	22769 (24.72)	515530 (26.26)
AG	8780 (11.07)	231352 (15.64)	12321 (9.82)	282280 (12.69)	12718 (13.21)	458946 (23.38)
AC	1670 (2.1)	28490 (1.93)	1968 (1.57)	32994 (1.48)	3400 (3.69)	62654 (3.19)
CG	6 (0.01)	86 (0.01)	4 (0.00)	62 (0.00)	6 (0.00)	86 (0.00)
Tri	10667 (13.44)	193242 (13.07)	13652 (10.88)	247191 (11.11)	12929 (14.04)	256068 (13.05)
AAG	3287 (4.14)	58542 (3.96)	4436 (3.54)	80292 (3.60)	4372 (4.75)	86421 (4.40)
AAT	1848 (2.33)	38862 (2.63)	2548 (2.03)	49839 (2.24)	2429 (2.64)	58134 (2.96)
AAC	1398 (1.76)	25053 (1.69)	1338 (1.07)	22425 (1.01)	1453 (1.58)	24603 (1.25)
ACC	720 (0.91)	11982 (0.81)	875 (0.70)	14634 (0.66)	819 (0.89)	13995 (0.71)
ACG	513 (0.65)	8619 (0.58)	520 (0.41)	8652 (0.39)	552 (0.60)	8991 (0.46)
AGG	1106 (1.39)	18759 (1.27)	1604 (1.28)	30024 (1.35)	1210 (1.31)	23403 (1.19)
ATC	1617 (2.04)	28551 (1.93)	2156 (1.72)	38541 (1.73)	1926 (2.09)	37851 (1.93)
CCG	1748 (0.22)	2874 (0.19)	175 (0.14)	2784 (0.13)	168 (0.18)	2670 (0.14)
Tetra	3684 (4.64)	65896 (4.46)	4884 (3.89)	86840 (3.90)	5832 (6.33)	178216 (9.08)
AAAC	390 (0.49)	6680 (0.45)	457 (0.36)	7856 (0.35)	428 (0.46)	7452 (0.38)
AAAG	446 (0.56)	7960 (0.54)	567 (0.45)	10240 (0.46)	564 (0.61)	10472 (0.53)
AAAT	1473 (1.86)	24908 (1.68)	1992 (1.59)	34120 (1.53)	1678 (1.82)	28808 (1.47)
AACT	148 (0.19)	2624 (0.18)	238 (0.19)	4220 (0.19)	147 (0.16)	2468 (0.13)
AATT	167 (0.21)	2800 (0.19)	458 (0.37)	9460 (0.43)	219 (0.24)	3712 (0.19)
ATAC	109 (0.14)	2088 (0.14)	126 (0.10)	2284 (0.10)	131 (0.14)	3648 (0.19)
ATAG	164 (0.21)	4992 (0.34)	304 (0.24)	5476 (0.25)	495 (0.54)	84172 (4.29)
Others	787 (0.99)	13844 (0.94)	742 (0.59)	13184 (0.59)	2170 (2.36)	37484 (1.91)
Penta	1341 (1.69)	28340 (1.92)	1781 (1.42)	37885 (1.70)	1713 (1.86)	37650 (1.92)
AAAAC	119 (0.15)	2515 (0.17)	145 (0.12)	3130 (0.14)	155 (0.17)	3400 (0.17)
AAAAT	234 (0.29)	4940 (0.34)	327 (0.26)	6900 (0.31)	316 (0.34)	6750 (0.34)
AACCG	199 (0.25)	4115 (0.28)	382 (0.30)	7885 (0.35)	157 (0.17)	3245 (0.17)
ACTGG	7 (0.01)	150 (0.01)	17 (0.01)	355 (0.02)	1 (0)	20 (0.00)
Others	782 (0.99)	16620 (1.12)	910 (0.73)	19615 (0.88)	1084 (1.18)	24235 (1.23)
Hexa	698 (0.88)	19932 (1.35)	1362 (1.09)	37452 (1.68)	1349 (1.46)	42990 (2.19)
AAAAAC	45 (0.06)	1440 (0.10)	58 (0.05)	1512 (0.07)	52 (0.06)	1770 (0.09)
AAAAAG	26 (0.03)	690 (0.05)	70 (0.06)	1806 (0.08)	198 (0.22)	5850 (0.30)
AAAAAT	85 (0.11)	2388 (0.16)	86 (0.07)	2244 (0.10)	84 (0.09)	2946 (0.15)
AAAACC	22 (0.03)	576 (0.04)	19 (0.02)	480 (0.02)	14 (0.02)	438 (0.02)
AAAGAG	15 (0.02)	402 (0.03)	20 (0.02)	522 (0.02)	16 (0.02)	402 (0.02)
AAATAT	21 (0.03)	1122 (0.08)	30 (0.02)	822 (0.04)	14 (0.02)	372 (0.02)
Others	484 (0.61)	13314 (0.90)	1079 (0.86)	30066 (1.35)	971 (1.05)	31212 (1.59)
Total	79341 (100)	1479031 (100)	125443 (100)	2225207 (100)	92089 (100)	1962930 (100)

**Table 2:** Distribution of the main SSR types in the genomes of the three allotetraploids.

Motif	*B. juncea*		*B. napus*		*B. carinata*	
	Number (%)	Total Length (%)	Number (%)	Total Length (%)	Number (%)	Total Length (%)
Mono	80518 (46.28)	1177653 (38.33)	79106 (45.57)	1173689 (31.13)	91679 (41.27)	1398206 (25.63)
A	77106 (44.32)	1117561 (36.38)	73785 (42.5)	1061674 (28.16)	89382 (40.23)	1368065 (25.08)
C	3412 (1.96)	60092 (1.96)	5321 (3.07)	112015 (2.97)	2297 (1.03)	30141 (0.55)
Di	56568 (32.52)	1185154 (38.58)	60145 (34.64)	1868030 (49.54)	84030 (37.82)	2815754 (51.32)
AT	29808 (17.13)	567802 (18.48)	37598 (21.66)	946490 (25.10)	54026 (24.32)	1841142 (33.75)
AG	22521 (12.95)	543962 (1.76)	19371 (11.16)	865648 (22.96)	24539 (11.05)	861550 (15.79)
AC	4226 (2.43)	73206 (2.38)	3162 (1.82)	55680 (1.48)	5450 (2.45)	112836 (2.07)
CG	13 (0.01)	184 (0.00)	14 (0.01)	212 (0.01)	15 (0.01)	226 (0.01)
Tri	23901 (13.74)	435792 (14.18)	22032 (12.69)	460536 (12.21)	26760 (12.05)	632148 (11.59)
AAG	8145 (4.68)	154041 (5.01)	7216 (4.16)	158442 (4.20)	8752 (3.94)	201657 (3.70)
AAT	3661 (2.10)	71178 (2.31)	4110 (2.37)	103179 (2.74)	5038 (2.27)	132789 (2.43)
AAC	2658 (1.53)	45213 (1.47)	2393 (1.38)	42348 (1.12)	2795 (1.26)	51114 (0.94)
ACC	1596 (0.92)	26676 (0.87)	1302 (0.75)	21654 (0.57)	1909 (0.86)	39012 (0.72)
ACG	1098 (0.63)	18114 (0.59)	952 (0.55)	15978 (0.42)	112 (0.05)	1803 (0.03)
AGG	2603 (1.50)	44328 (1.44)	2109 (1.21)	36276 (0.96)	2740 (1.23)	60408 (1.11)
ATC	3822 (2.20)	71109 (2.31)	3631 (2.09)	77541 (2.06)	4091 (1.84)	104343 (1.91)
CCG	318 (0.18)	5133 (0.17)	319 (0.18)	5118 (0.14)	394 (0.18)	23763 (0.44)
Tetra	8570 (4.93)	166296 (5.41)	7791 (4.49)	157596 (4.18)	11798 (5.31)	279644 (5.13)
AAAC	908 (0.52)	15660 (0.51)	833 (0.48)	14284 (0.38)	862 (0.39)	17548 (0.32)
AAAG	1068 (0.61)	19532 (0.64)	927 (0.53)	17536 (0.47)	1157 (0.52)	22680 (0.42)
AAAT	2860 (1.64)	48480 (1.58)	3179 (1.83)	54672 (1.45)	3777 (1.7)	74496 (1.37)
AACT	284 (0.16)	5000 (0.16)	453 (0.22)	8052 (0.21)	75 (0.03)	1756 (0.03)
AATT	337 (0.19)	5812 (0.19)	456 (0.26)	9724 (0.26)	776 (0.35)	22872 (0.42)
ATAC	260 (0.15)	5680 (0.18)	215 (0.12)	4156 (0.11)	259 (0.12)	7848 (0.15)
ATAG	934 (0.54)	33156 (1.08)	469 (0.27)	26228 (0.70)	1570 (0.71)	61660 (1.13)
Others	1919 (1.12)	32976 (1.07)	1259 (0.73)	22944 (0.61)	3322 (1.49)	70784 (1.30)
Penta	2739 (1.57)	59810 (1.95)	2770 (1.60)	60990 (1.62)	4238 (1.91)	134265 (2.46)
AAAAC	295 (0.17)	6420 (0.21)	271 (0.16)	5800 (0.15)	276 (0.12)	6030 (0.11)
AAAAT	518 (0.30)	10915 (0.36)	530 (0.31)	11405 (0.30)	716 (0.32)	19415 (0.36)
AACCG	386 (0.22)	8095 (0.26)	508 (0.29)	10445 (0.28)	671 (0.30)	18425 (0.34)
ACTGG	30 (0.02)	630 (0.02)	49 (0.03)	1005 (0.03)	23 (0.01)	475 (0.01)
Others	1510 (0.86)	33750 (1.10)	1412 (0.84)	32335 (0.86)	2552 (1.16)	89920 (1.65)
Hexa	1668 (0.96)	47610 (1.55)	1760 (1.01)	49902 (1.32)	3655 (1.65)	195090 (3.58)
AAAAAC	134 (0.08)	3960 (0.13)	99 (0.06)	2730 (0.07)	118 (0.05)	3306 (0.06)
AAAAAG	159 (0.09)	4236 (0.14)	80 (0.05)	2034 (0.05)	243 (0.11)	16302 (0.30)
AAAAAT	118 (0.07)	3048 (0.10)	159 (0.09)	4332 (0.11)	327 (0.15)	15234 (0.28)
AAAACC	28 (0.02)	786 (0.03)	23 (0.01)	636 (0.02)	34 (0.02)	2394 (0.04)
AAAGAG	30 (0.02)	822 (0.03)	34 (0.02)	912 (0.02)	44 (0.02)	1200 (0.02)
AAATAT	15 (0.01)	432 (0.01)	36 (0.02)	1626 (0.04)	74 (0.03)	3558 (0.07)
Others	1184 (0.67)	34326 (1.12)	1329 (0.77)	37632 (1.00)	2815 (1.27)	153096 (2.81)
Total	173694 (100)	3072315 (100)	173604 (100)	3770743 (100)	222160 (100)	5455107 (100)

**Figure 2:**
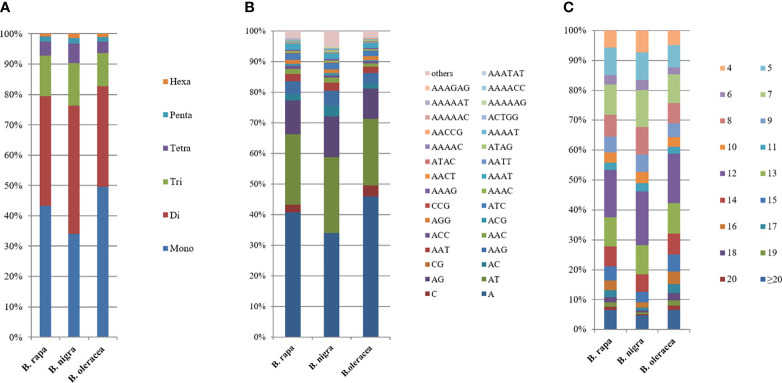
Distribution with respect to the motif length **(A)**, type **(B)** and repeat number **(C)** of microsatellites in the whole genomes of *B. rapa, B. nigra* and *B. oleracea*. The vertical axis represents the abundance (%) of microsatellites of different motif lengths, types or number of repeats, which are distinguished by different colors. For **(B)**, due to the limited number of items in Excel, the abundance of representative single to pentanucleotide motifs was selected, while the abundance of other motifs was shown in **Supplementary Table 2**.

In the genomic SSRs of the six studied species, the distribution of microsatellite motif lengths was almost identical except in *B. nigra* (BB); mononucleotide, dinucleotide, trinucleotide and tetranucleotide repeats accounted for a very similar and relatively high proportion, while pentanucleotide and hexanucleotide repeats were relatively uncommon. The mononucleotide repeat motifs were the most abundant of the repeat types, with frequencies largely above 40%, and even close to 50% in *B. oleracea* (**Figure 2A**).

The type distribution of microsatellite motifs was almost identical in the whole genome sequences of *B. rapa*, *B. nigra*, *B. oleracea*, *B. napus*, *B. juncea*, and *B. carinata* (**Figure 2B**). In other words, the mononucleotide to hexanucleotide motifs making up the major part of the genome sequences of the six *Brassica* species and those that are scarce were essentially the same. From **Figure 2B**, it is interesting to find that among the mononucleotide repeat sequences, A has the most repetitive motifs; among the dinucleotide repeat sequences, AT has the most repetitive motifs, followed by AG; among the trinucleotide repeat sequences, AAG has the most repetitive sequences, followed by AAC; among the tetranucleotides AAAT has the most repetitive sequences; among the five and six nucleotides, AAAAT and AAAAAT are also more common than other combinations. Most of the single to hexanucleotide sequences that account for the major motifs contained abundant A\T, while the G\C motifs are all among the scarce motifs. This is in good agreement with previous reports of microsatellites identified in *B. rapa, B. oleracea* and *B. napus.* It is also clearly seen that the genomic sequences of *B. rapa* have a much higher content of A\T relative to C\G.

**Figure 3:**
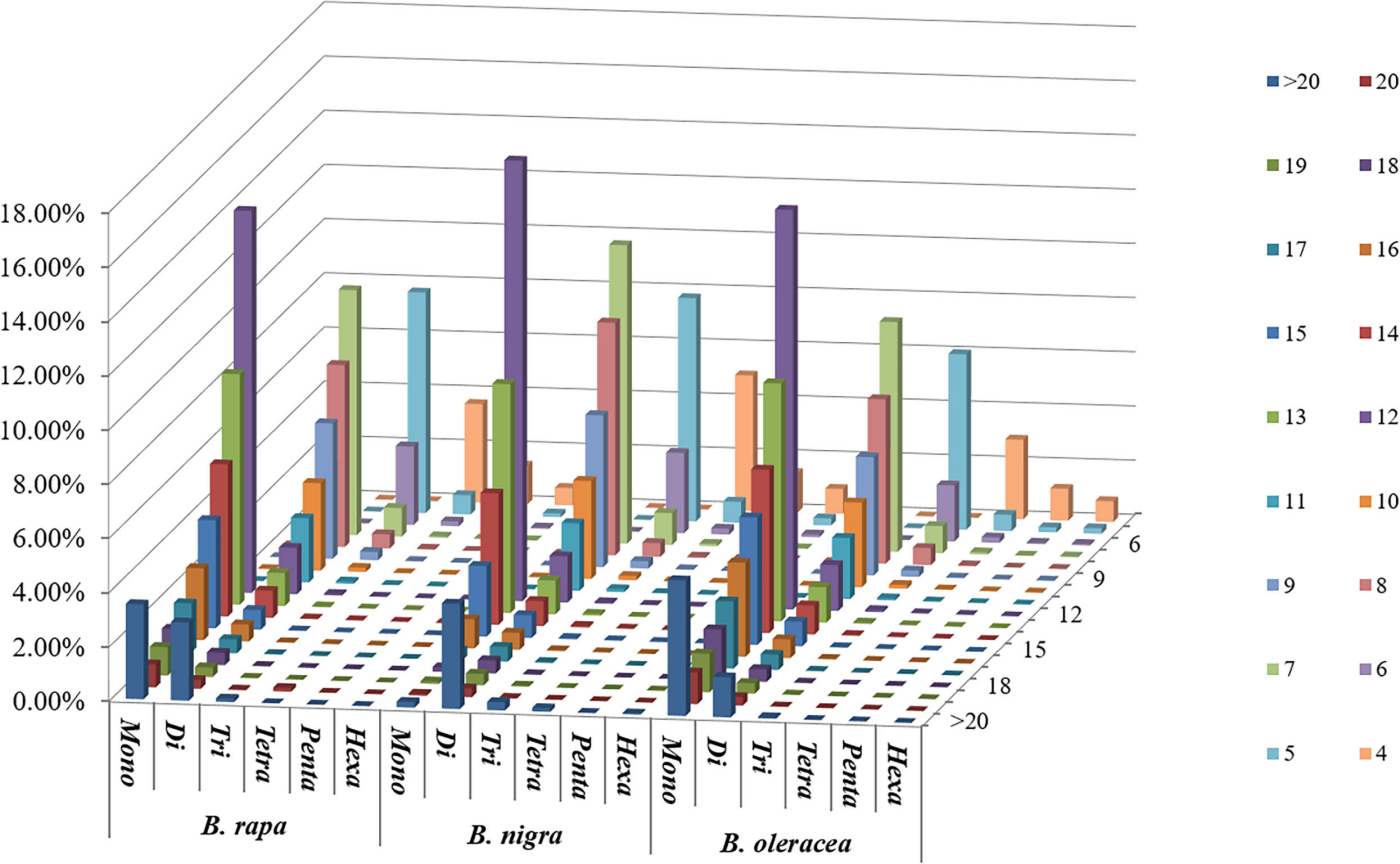
Distribution with respect to the motif repeat number of the individual mono- to hexanucleotide repeat microsatellites in the whole genomes of *B. rapa*, *B. nigra* and *B. oleracea*. The vertical axis shows a large number of microsatellites with different motif repeat numbers (from 4 to 20), which are distinguished by different colors.

**Figure 4:**
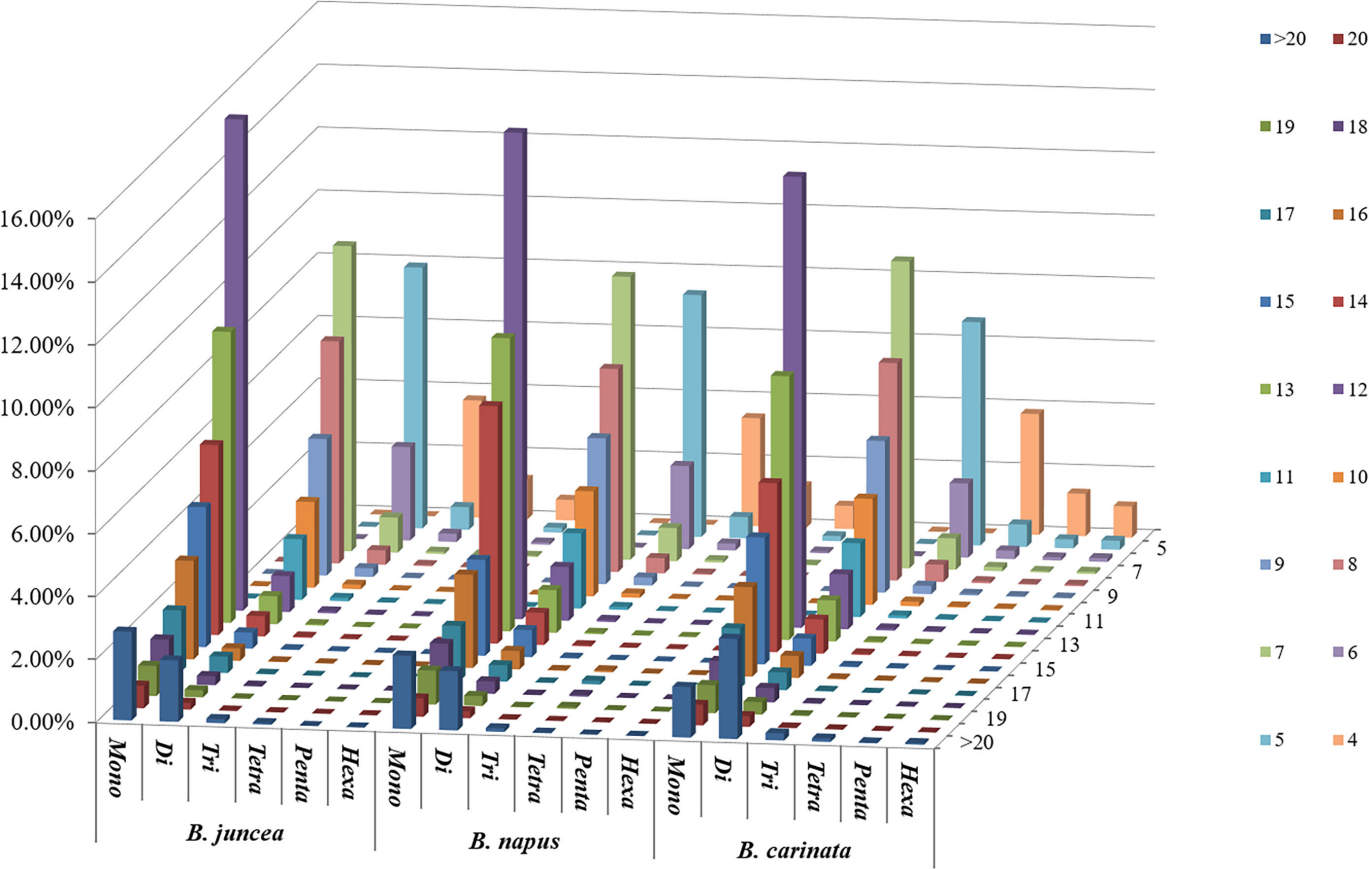
Distribution with respect to the motif repeat number of the individual mono- to hexanucleotide repeat microsatellites in the whole genomes of *B. juncea*, *B. napus* and *B. carinata*. The vertical axis shows a large number of microsatellites with different motif repeat numbers (from 4 to 20), which are distinguished by different colors.

Among the whole genome sequences of *B. rapa* (AA), *B. nigra*, *B. oleracea*, *B. napus*, *B. juncea*, and *B. carinata*, the distribution pattern of the number of motif repeats of microsatellites is essentially the same, except for *B. nigra*, where 12 repeats have the highest proportion of all repeats. (**Figure 2C**). At the same time, we can see that the microsatellite abundance decreases significantly as the number of motif repeats increases, with the rate of change being the flattest for dinucleotides, followed by single nucleotide as well as trinucleotide repeats, and more drastic changes can be observed for long repeat motifs. (**Figures 3**, **4**).

**Figure 5:**
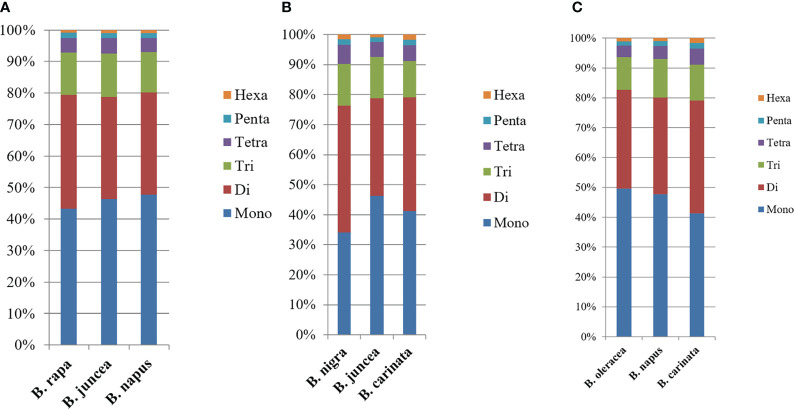
Distribution with respect to the motif length of microsatellites in the whole genomes of *Brassica* species in the triangle of U. **(A)** Distribution with respect to the motif length of microsatellites in the whole genomes of *B. rapa*, *B. napus* and *B. juncea.* **(B)** Distribution with respect to the motif length of microsatellites in the whole genomes of *B. nigra*, *B. juncea* and *B. carinata.* **(C)** Distribution with respect to the motif length of microsatellites in the whole genomes of *B. oleracea*, *B. napus* and *B. carinata*. The vertical axis shows the abundance (%) of microsatellites with different motif lengths, which are distinguished by different colors.

In addition, we compared the corresponding motif lengths (**Figure 5**), mono- to hexanucleotide microsatellite numbers and motif repeat numbers between the basic species and the two heterotetraploid variants from which they diverged. As can be seen from the figure, the patterns of variation are very similar in the genomic SSRs of the six studied species, but comparisons between them reveal a more similar trend in microsatellite length distribution between *B. rapa* (AA), *B. napus* (AACC) and *B. juncea* (AABB) (**Figure 5A**); among *B. nigra* (BB), *B. juncea* (AABB) and *B. carinata* (BBCC), the microsatellite length distribution trends are more similar between *B. nigra* (BB) and *B. carinata* (BBCC) (**Figure 5B**); whereas among *B. oleracea* (CC), *B. napus* (AACC) and *B. carinata* (BBCC), the microsatellite length distribution trends are more similar between *B. oleracea* (CC) and *B. napus* (AACC) (**Figure 5C**). However, these differences are not highly significant.

By comparing the number distribution of the three allotetraploid SSR loci in the three subgenomes AA, BB and CC, we can find that the allotetraploid species have significant differences in the number of SSR loci in the genome compared with their basic diploid counterparts (**Table 3**). It is worth mentioning that the reduction in SSR loci is the highest during the allotetraploidization of *B. napus* (AACC), which is 31.3% and 37%, respectively, compared with the diploid AA and CC genome. Furthermore, the reduction in SSR loci is intermediate during the allotetraploidization of *B. juncea* (AABB) L, which is 7.3% and 13.0%, respectively, compared with the diploid AA and BB genome. Moreover, the reduction in SSR loci is the lowest during the allotetraploidization of *B. carinata* (BBCC) L, which is 1.7% and 6.0%, respectively, compared with the diploid BB and CC genome (**Table 3**). We also compared the distribution of subgenomic chromosome SSR loci in the three basic species and the three allotetraploids respectively, and found that although the number of SSR loci in most chromosomes showed a downward trend, a few chromosomes showed an increase (**Supplementary Table 1**). For example, in *B. juncea*, the A subgenomic chromosomes A01 and A02 increased by 5.55% and 2.60% compared with the basic species, respectively. Moreover, in *B. carinata*, the B subgenomic chromosomes B01, B03 and B04 increased by 49.53%, 11.17% and 3.13%, respectively (**Supplementary Table 1**). These results suggest that the cytological and genetic mechanisms of allotetraploid evolution are complex and worthy of further study in the future.

**Table 3:** The number of SSR loci in different genomes in the *B Brassica* species in the triangle of U.

Species	AA genome/subgenome	BB genome/subgenome	CC genome/subgenome
*B rapa* (AA)	79341	0	0
*B nigra* (BB)	0	92089	0
*B oleracea* (CC)	0	0	125443
*B juncea* (AABB)	73584 (7.3% ↓)	80149 (13.0% ↓)	0
*B napus* (AACC)	54500 (31.3% ↓)	0	79084 (37.0% ↓)
*B carinata* (BBCC)	0	90569 (1.7% ↓)	117871 (6.0% ↓)

“↓” means reducing of the number SSR loci.

### 3.2 Distribution and physical location of SSRs on each chromosome of the whole genome of *Brassica* species in the triangle of U

Based on sequencing the whole genome chromosomes of *Brassica* species in the triangle of U, the characteristics of microsatellites on each chromosome of each of the six considered *Brassica* species and their physical location were investigated.

The characteristics of length, type and number of repeats on each chromosome of the six *Brassica* species were consistent with the overall microsatellite characteristics of each species described above. However, the number of microsatellites distributed on each chromosome was extremely heterogeneous. For the basic species *B. rapa* (AA) (**Supplementary Table 2**), *B. nigra* (BB) (**Supplementary Table 3**) and *B. oleracea* CC (**Supplementary Table 4**), the number of microsatellites was the highest on A09 (11214), B02 (13909) and C03 (18286), respectively. In contrast, for the four allotetraploid variants *B. napus* (AACC) (**Supplementary Table 5**), *B. juncea* (AABB) (**Supplementary Table 6**) and *B. carinata* (BBCC) (**Supplementary Table 7**), the microsatellite numbers were the highest on C03 (12561), B02 (12452) and C01 (16036), respectively. This may have occurred because the number of microsatellites is closely related to the length of the chromosomes; the greater the length of a chromosome, the larger the number of its corresponding microsatellites.

In order to explore the exact distribution of microsatellites on each chromosome, the relationship between microsatellites and chromosomes and that between the three basic species and their corresponding allotetraploids should be analyzed more clearly. We used mapping software to locate the physical position of each microsatellite to the corresponding chromosome. The results show that, for the six *Brassica* species, *B. rapa* (AA), *B. nigra* (BB), *B. oleracea* (CC), *B. juncea* (AABB), *B. napus* (AACC), and *B. carinata* (BBCC), all chromosomes have higher microsatellite frequencies at and near the ends, and they present lower microsatellite frequencies in and near the middle region. This is consistent with previous studies on the location of microsatellites on chromosomes and may correspond to the distribution around the telomeres and the thylakoids. Secondly, the physical distribution of microsatellites across all chromosomes of the six different species of *Brassica* is highly heterogeneous, suggesting that microsatellites do not occur randomly but their presence is most likely highly correlated with gene function around them. By comparison, we can also find that microsatellite distribution is more concentrated in *B. nigra* (BB) based on the physical position of microsatellites among the three basic species. Meanwhile, in the three allotetraploids, microsatellite distribution is more concentrated in *B. carinata* (BBCC), which may be due to the more concentrated distribution of genes on *B. nigra* (BB) and *B. carinata* (BBCC). The more concentrated distribution of genes on the latter two species is probably related. Moreover, the high concordance between microsatellites and genes strongly suggests the putative role of microsatellites in regulating genome function and in tagging genes using SSR molecules.

### 3.3 SSR primer design of the whole genome of Br*assica* species in the triangle of U

Using Krait software, primer pairs were successfully designed for each of the six species of *B. rapa* (AA), *B. nigra* (BB), *B. oleracea* (CC), *B. napus* (AACC), *B. juncea* (AABB) and *B. carinata* (BBCC), respectively, yielding a total of 52356, 62290, 82984, 111276, 120324 and 144149 primer pairs named in the order of *BrSSR00001* ~ *BrSSR52356*, *BniSSR00001* ~ *BniSSR62290*, *BolSSR000001* ~ *BolSSR082984*, *BnaSSR000001* ~ *BnaSSR111276*, *BjuSSR000001* ~ *BjuSSR120324*, and *BcaSSR000001* ~ *BcaSSR144149*, respectively. The primer sequence, TM value, SSR motif, expected product length, and start/end position on the chromosome for each SSR marker were determined (**Supplementary Tables 8**–**13**).

### 3.4 Transferability evaluation of the whole genome of B*rassica* species in the triangle of U

In this study, to enrich the SSR marker library of cruciferous crops and confirm the validity of the designed SSR primers, we randomly selected 120 primer pairs (**Supplementary Tables 14**–**16**) in three basic species and used genomic DNA from eight cruciferous species as DNA template, namely, *B. rapa* (AA), *B. nigra* (BB), *B. oleracea* (CC), *B. juncea* (AABB), *B. napus* (AACC), *B. carinata* (BBCC), *R. sativus* (RR), and *A. thaliana* (At). The cross-transferability of SSR markers from *B. rapa* (40 SSRs), *B. nigra* (40 SSRs) and *B. oleracea* (40 SSRs) was assessed and the affinities of the three basic species in forming three allotetraploids were speculated. We used a total of 120 SSRs, 40 from *B. rapa*, 40 from *B. nigra*, and 40 from *B. oleracea* for cross-amplification studies.

For the 40 primer pairs of *B. rapa*, a total of 310 positive amplifications were made in the eight species, resulting in a total of 325 alleles amplified with a cross-transfer rate of 85.27% (**Table 4** and **Supplementary Figure 1A**). This high cross-transfer rate presumes the validity of these SSR markers for the study of the genomes of other species of cruciferous crops. Among these 325 amplified markers, the PIC values ranged from 0.32 to 0.84 with a mean value of 0.73. The gene diversity ranged from 0.41 to 0.86 with a mean value of 0.7461, and the heterozygosity values ranged from 0.21 to 0.70 with a mean value of 0.55.

**Table 4:** Amplification results of the *B. rapa* cross-transferability test.

SSR	*B. rapa*	*B. nigra*	*B. oleracea*	*B. juncea*	*B. napus*	*B. carinata*	*A. thaliana*	*R. sativus*
BrSSR00004	**+**	**+**	**-**	**+**	**+**	**+**	**+**	**+**
BrSSR01587	**+**	**+**	**+**	**+**	**-**	**-**	**+**	**+**
BrSSR03830	**+**	**+**	**+**	**+**	**+**	**+**	**+**	**+**
BrSSR03677	**+**	**+**	**+**	**+**	**+**	**+**	**+**	**+**
BrSSR01972	**+**	**+**	**+**	**+**	**+**	**+**	**+**	**+**
BrSSR30590	**+**	**+**	**+**	**+**	**+**	**+**	**+**	**+**
BrSSR06092	**+**	**+**	**+**	**+**	**+**	**+**	**-**	**-**
BrSSR09075	**+**	**+**	**+**	**+**	**+**	**+**	**-**	**+**
BrSSR14973	**+**	**+**	**-**	**+**	**+**	**+**	**+**	**+**
BrSSR15994	**+**	**+**	**+**	**+**	**+**	**+**	**-**	**-**
BrSSR13664	**+**	**+**	**-**	**+**	**+**	**-**	**-**	**-**
BrSSR11198	**+**	**-**	**+**	**+**	**+**	**+**	**+**	**+**
BrSSR17217	**+**	**+**	**-**	**+**	**+**	**+**	**+**	**-**
BrSSR18858	**+**	**+**	**+**	**+**	**+**	**-**	**-**	**-**
BrSSR16468	**+**	**+**	**+**	**+**	**+**	**+**	**+**	**+**
BrSSR18217	**+**	**+**	**+**	**+**	**+**	**+**	**+**	**+**
BrSSR18436	**+**	**+**	**+**	**+**	**+**	**+**	**+**	**+**
BrSSR21863	**+**	**+**	**+**	**+**	**+**	**+**	**+**	**+**
BrSSR23017	**+**	**+**	**+**	**+**	**+**	**+**	**+**	**+**
BrSSR24442	**+**	**+**	**+**	**+**	**+**	**+**	**+**	**+**
BrSSR28675	**+**	**-**	**+**	**+**	**-**	**-**	**-**	**-**
BrSSR25854	**+**	**+**	**+**	**+**	**+**	**-**	**-**	**-**
BrSSR31751	**+**	**+**	**+**	**+**	**+**	**+**	**-**	**-**
BrSSR34080	**+**	**+**	**-**	**+**	**+**	**-**	**+**	**+**
BrSSR33336	**+**	**+**	**+**	**+**	**+**	**-**	**+**	**-**
BrSSR34987	**+**	**+**	**+**	**+**	**+**	**+**	**+**	**-**
BrSSR36313	**+**	**+**	**-**	**+**	**+**	**-**	**-**	**-**
BrSSR37152	**+**	**+**	**-**	**+**	**+**	**-**	**-**	**-**
BrSSR37224	**+**	**+**	**+**	**+**	**+**	**+**	**+**	**-**
BrSSR36383	**+**	**+**	**+**	**+**	**+**	**+**	**+**	**+**
BrSSR38719	**+**	**-**	**-**	**-**	**+**	**-**	**-**	**-**
BrSSR39889	**+**	**+**	**-**	**+**	**+**	**-**	**+**	**-**
BrSSR42556	**+**	**+**	**+**	**+**	**+**	**+**	**+**	**+**
BrSSR44040	**+**	**-**	**+**	**+**	**+**	**+**	**-**	**-**
BrSSR44329	**+**	**+**	**-**	**+**	**+**	**-**	**-**	**-**
BrSSR45708	**+**	**+**	**+**	**+**	**+**	**+**	**-**	**-**
BrSSR47369	**+**	**+**	**+**	**+**	**+**	**+**	**+**	**+**
BrSSR48533	**+**	**+**	**-**	**+**	**+**	**+**	**+**	**-**

+ means positive result which show the expectant amplification band; - means negative result which show none of the expectant amplification band.

For the 40 primer pairs of *B. nigra*, a total of 280 positive amplifications were made across the eight species, resulting in a total of 301 alleles amplified with a cross-transfer rate of 81.33% (**Supplementary Table 17** and **Supplementary Figure 1B**). From this high rate of cross-transfer, we speculate that these SSR markers have some validity for genomic studies of other species of cruciferous crops. The PIC values of these 301 amplified markers ranged from 0.33 to 0.73 with a mean value of 0.50. The gene diversity ranged from 0.38 to 0.71 with a mean value of 0.58, and the heterozygosity values ranged from 0.10 to 0.53 with a mean value of 0.41.

For the 40 primer pairs of *B. oleracea*, a total of 303 positive amplifications were made across the eight species, resulting in a total of 310 amplified alleles with a cross-transfer rate of 73.45% (**Supplemntary Table 18** and **Supplementary Figure 1C**). This indicates that the SSR cross-transfer rate of *B. oleracea* has limitations in terms of its validity for studying the genomes of other species of cruciferous crops. Among these 310 amplified markers, the PIC values ranged from 0.48 to 0.85 with a mean of 0.67. The gene diversity ranged from 0.54 to 0.81 with a mean of 0.64, and the heterozygosity values ranged from 0.0 to 0.71 with a mean of 0.41.

When comparing the polymorphism potential of *B. rapa* (85.27%), *B. nigra* (81.33%) and *B. oleracea* (73.45%), *B. rapa* was found to have a higher cross-transfer rate (85.27%). In addition, the mean values of PIC, genetic diversity and heterozygosity of *B. rapa*-derived SSR markers were relatively high, indicating that among cruciferous plant species, *B. rapa* has better polymorphic potential than *B. nigra* and *B. oleracea*. The cross-species transferability has been demonstrated in *Brassica* crops, while the degree of SSR cross-transfer depends on the evolutionary distance among species (Thakur et al., 2022).

### 3.5 Application of SSR molecular markers of *Brassica* species in the triangle of U

**Figure 6:**
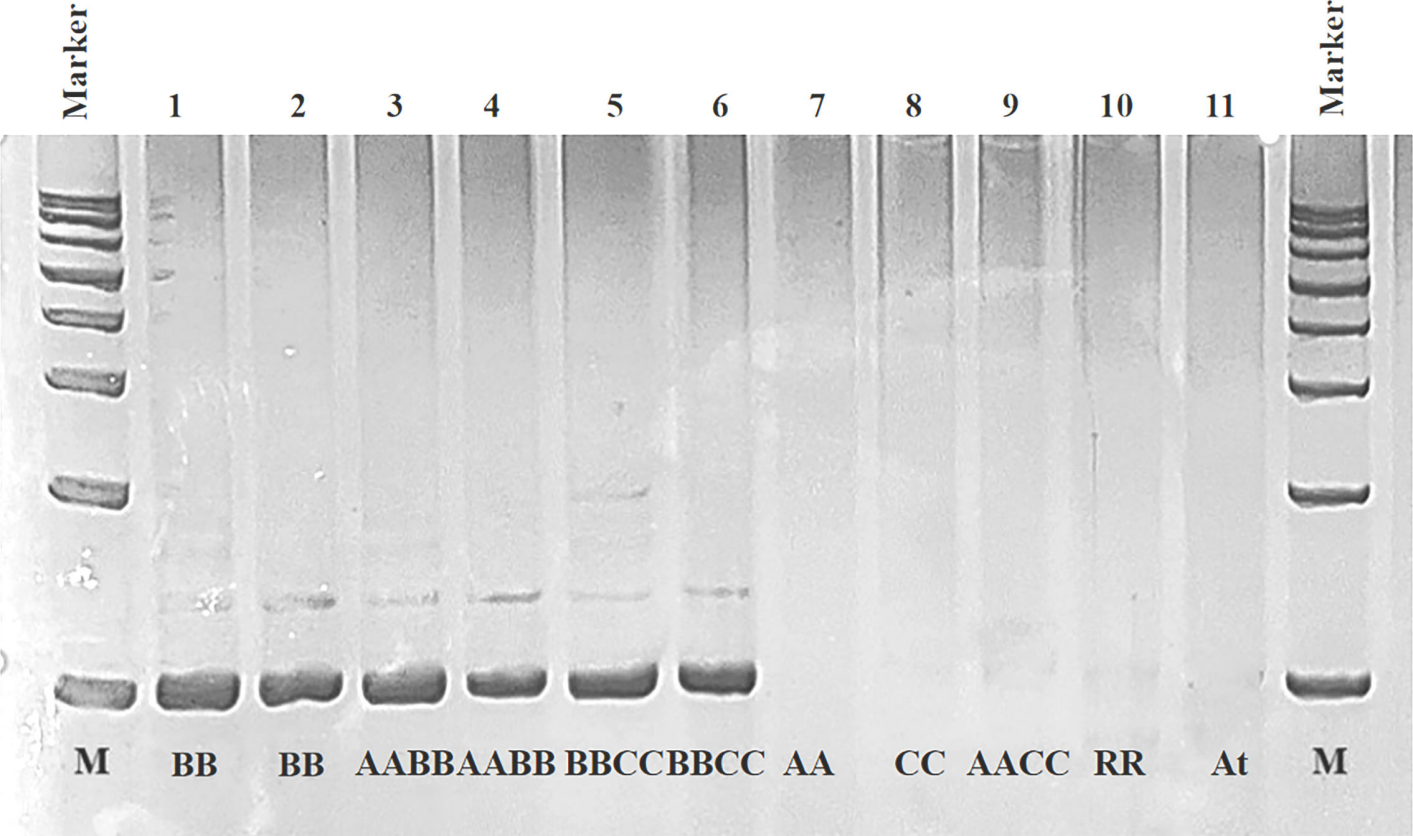
PCR amplification result of validity for *BniSSR23228* in different *Brassica* species in the triangle of U. 1, 2: *B. nigra* (BB); 3, 4: *B. juncea* (AABB); 5, 6: *B. carinata* (BBCC); 7, 8, 9, 10, and 11: *B. rapa* (AA), *B. oleracea* (CC), *B. napus* (AACC), *R. sativus* (RR) and *A. thaliana* (At), respectively.

A B-genome specific SSR marker, *BniSSR23228*, was obtained from 40 selected SSR primers of black mustard (**Supplementary Figure 1B**). After PCR amplification, polyacrylamide gel electrophoresis, cloning verification screening and sequence alignment were carried out to validate the existence of this specific SSR marker (**Figure 6**). The validity experiment results indicated that *BniSSR23228* possessing (AAGGA)_3_ sequence characteristics located in chromosome B3 with a total length of 97 bp (**Supplementary Figure 2**). Subsequently, this molecular marker can effectively screen the B genome of *Brassica*, and can be used for variety and parent identification, introgression line tracking, genetic diversity analysis, and marker-assisted breeding.

## 4 Discussion

### 4.1 Distribution feature of the SSR in the whole genome of *Brassica*

Krait software was used to search for 79341, 92089, 125443, 173964, 173604, and 222160 SSR loci from the whole genome sequences of six species of *Brassica*, namely, *B. rapa* (AA), *B. nigra* (BB), *B. oleracea* (CC), *B. napus* (AACC), *B. juncea* (AABB), and *B. carinata* (BBCC), respectively. The frequency of SSR occurrences (average SSRs per Mb) was 226 loci/Mb, 182 loci/Mb, 223.6 loci/Mb, 231.31 loci/Mb, 235.12 loci/Mb and 204.42 loci/Mb, respectively. In a previous report, the PERL5 script MIcroSAtellite (MISA; http://pgrc.ipkgatersleben.de/misa/) was employed for the genomes of *B. rapa*, *B. oleracea* and *B. napus* to obtain 140998, 229389 and 420991 SSR markers, respectively (Shi et al., 2014). Using Karit enabled SSR identification and subsequent primer design in less time than MISA, and the long microsatellites identified in this way were more polymorphic and useful. Among the SSR single nucleotide sequences identified in the six species, *B. rapa* (AA), *B. nigra* (BB), *B. oleracea* (CC), *B. napus* (AACC), *B. juncea* (AABB), and *B. carinata* (BBCC), the A sequence repeats were present in 29518 (42.5%), 31308 (34.00%), 57629 (45.94%), 73785 (42.5%), 77106 (44.32%), and 89382 (40.23%) single nucleotides, respectively. They were therefore regarded as the most important of such repeats, while the C sequence was less represented in single nucleotides. This result is consistent with previous studies performing SSR analysis of the whole grapevine genome (Cai et al., 2009). For the dinucleotide repeat type AT, the number of repeats were 13915 (20.04%), 22769 (24.72%), 27267 (21.74%), 37598 (21.66%), 29808 (17.13%) and 54026 (24.32%) respectively. This is therefore considered as the most significant of this replicate type, consistent with previous studies using SSR analysis of the whole B73 maize genome (Qu and Liu, 2013). The frequency of single nucleotide repeats was the highest of all repeat types, a result that differs from previous SSR analysis studies using MISA in *B. rapa*, *B. oleracea* and *B. napus* (Shi et al., 2014). Moreover, Shi et al. study results showed the microsatellite frequencies of *Brassica*, *Arabidopsis* and other angiosperm species were significantly negatively correlated with both their genome sizes and transposable elements contents (Shi et al., 2013). Qin et al. (2015) investigated the evolutionary regularities of SSRs during the evolution of plant species and the plant kingdom by analysis of twelve sequenced plant genome sequences. The results showed that, SSRs not only had the general pattern in the evolution of plant kingdom, but also were associated with the evolution of the specific genome sequence.

Probably, the deviation may be due to differences in the SSR software algorithms, parameter settings and the original databases used. In addition, our data indicated that the allotetraploidization process resulted in a significant reduction in SSR loci in the three subgenomes AA, BB and CC. The reasons may be partial gene-dominated chromosomal homologous recombination and rearrangement during the evolution of basic diploid species into allotetraploids (Song et al., 2021). Meanwhile, there are a large number of transposable elements (TE) in the *Brassica* species in the triangle of U genome (Cai et al., 2022), and TE insertion seems to result in chromosomal translocation, leading to the reduced number of SSR loci in the three subgenomes AA, BB and CC during the process of allotetraploidization. Of course, further experiments are required to prove this hypothesis. Shi et al. (2014) carried out microsatellite characterization based on genome-wide and marker development in three recently sequenced *Brassica* crops, and suggested that the distribution pattern of microsatellites may be conserved in the genus *Brassica*. This view was reinforced by the use of Krait to identify SSR signatures of six species of *Brassica*, suggesting that the distribution patterns of microsatellites are likely to be conserved in all *Brassica* species. Thus far, the comprehensive identification, characterization and primer development of SSRs for six *Brassica* species in the triangle of U have not been carried out. However, in this study, based on the complete whole genome sequences of six *Brassica* species in the triangle of U, not only were the SSRs of each variety comprehensively analyzed, but also the differences between the *Brassica* crops in the triangle of U were compared and a comprehensive primer design for the SSRs was carried out. To our knowledge, this is the first report to identify the SSR loci and design the SSR primers based on the complete whole genome sequences of six *Brassica* species in the triangle of U together. These markers will act as a powerful tool for future genomic and genetic studies of *Brassica* cruciferous crops in the near future.

### 4.2 Enrichment of the repertoire of SSR markers of *Brassica* using the cross-transferability approach

High transferability has been reported for SSRs of different plant species, such as “Chiifu” of *B. rapa*, that is, 95% of its SSRs could amplify a fragment of other species (Wang et al., 2011). Thakur et al. (2018) study result indicated 100% cross-transferability was obtained for *B. juncea* and three subspecies of *B. rapa* with 124 *Brassica*-derived SSR loci assayed, while lowest cross-transferability (91.93%) was obtained for *Eruca sativa*. The average % age of cross-transferability across all the seven species was 98.15%. In addition, 47% of EST-SSR markers developed from *B. rapa*, *B. oleracea*, and *B. napus* were transferable to six *Brassica* species (An et al., 2011). Sim et al. randomly selected 41 SSR markers of thistle and alfalfa, and found that the transferability was 53% to 71% in the leguminous plant (alfalfa) and 33% to 44% in the non-leguminous plant (thistle). About 57% of cereal EST-SSRs could also be amplified in ryegrass (Sim et al., 2009). Additionally, about 60% of EST-SSR markers from barley could be amplified in wheat and rye (Castillo et al., 2008). Cui et al. (2005) used 69 pairs of SSR primers of non-heading Chinese cabbage in eight varieties of *Brassica* crops, and found that the transferability amplification rate was 49.3% to 85.5% and that 33% of the SSR primers in the inter-specific hybrids of *Brassica* presented abundant diversity.

Based on the 1176 SSR-containing ESTs in cabbage, a total of 978 primer pairs have been successfully designed and assessed by validation of the amplification on two inbreed lines (Chen et al., 2010). Subsequently, the results indicated that the developed SSRs from ESTs of *B. oleracea* were valid and practicable in marker-assisted selection and QTL analysis in cabbage (Su et al., 2015). Some useful information about SSR and sequence analysis in *Brassica* crops can also be obtained on the website of *Brassica* DB database (http://Brassica.bbsrc.ac.uk/).

In this work, the functional utility of SSR markers derived from *B. rapa* (AA), *B. nigra* (BB) and *B. oleracea* (CC) was evaluated by analyzing their cross-transferability among *B. rapa* (AA), *B. nigra* (BB), *B. oleracea* (CC), *B. juncea* (AABB), *B. napus* (AACC), *B. carinata* (BBCC), *R. sativus* (RR), and *A. thaliana* (At). From our results, it was inferred that the cross-transferability of SSR markers from *B. rapa* (AA) showed higher potential than those from *B. nigra* (BB) and *B. oleracea* (CC) among these eight species, with cross-transferability rates of 85.27%, 81.33% and 73.45%, respectively. In fact, enriching other varieties with SSR markers alleviates the hassle of the expansion and development process and can facilitate the genetic improvement of new varieties by the genomes of superior varieties. Our findings suggest that genomic SSR markers with high transferability can be used for different *Brassica* species and even non-*Brassica* species. Therefore, these genomic SSR markers with clear location and uniform nomenclature system have high potential to be more widely used in several fields, such as gene localization, genetic mapping, evolutionary analysis, molecular marker-assisted breeding, and provide marker materials for genetic and comparative genomics analysis to further introduce some important agronomic traits into other superior *Brassica* species lacking these traits.

### 4.3 *BniSSR23228* is a B-genome specific SSR marker

Alien chromosome additions have been used to link species-specific characteristics to particular chromosomes (Kapoor et al., 2011). The plasticity of the *Brassica* genome and existence of natural amphiploids have made it possible to develop several alien chromosome additions by dissecting the *B. rapa*, *B. oleracea*, and *B. nigra* genomes (Chevre et al., 1996; Gu et al., 2009; Li et al., 2013). Compared with the single species-specific SSR marker obtained by comparison between two species developed in previous studies (Gu et al., 2009; Li et al., 2013), the specific SSR developed in this study, *BniSSR23228*, has been verified among *Brassica* species in the triangle of U and their closely related species, radish and *Arabidopsis*. The results revealed that it is a B-genome specific SSR marker, therefore has more significant B-genome specificity and more extensive application value in the future.

## Data availability statement

Publicly available datasets were analyzed in this study. This data can be found here: http://brassicadb.cn.

## Author contributions

NS: Methodology, Software, Validation, Writing – original draft, Writing – review & editing. JC: Conceptualization, Funding acquisition, Resources, Supervision, Writing – review & editing. YW: Formal analysis, Investigation, Methodology, Validation, Writing – review & editing. IH: Data curation, Validation, Writing – review & editing. NL: Funding acquisition, Resources, Validation, Writing – review & editing. XM: Data curation, Investigation, Validation, Writing – review & editing. WL: Data curation, Formal analysis, Investigation, Writing – review & editing. KL: Data curation, Validation, Visualization, Writing – review & editing. HY: Formal analysis, Methodology, Visualization, Writing – review & editing. KZ: Data curation, Investigation, Validation, Visualization, Writing – review & editing. TZ: Data curation, Formal analysis, Investigation, Writing – review & editing. YZ: Data curation, Software, Validation, Writing – review & editing, Writing – original draft. XY: Conceptualization, Funding acquisition, Project administration, Resources, Supervision, Writing – review & editing, Writing – original draft.

## Supplementary material

Supplementary Figure 1 Transferability analysis on the designed SSR primers for the three basic species. **(A)**, PCR amplification results of SSR primers for part of the AA genome; **(B)**, PCR amplification results of SSR primers for part of the BB genome; C, PCR amplification results of SSR primers for part of the CC genome.

Supplementary Figure 2 Genomic sequence of *BniSSR23228* and sequence alignment results for the BB, AA and CC genomes. **(A)**, Genomic sequence of *BniSSR23228*; **(B)**, Sequence alignment result for the BB genome; **(C)**, Sequence alignment result for the AA genome; **(B)**, Sequence alignment result for the CC genome.
