# Supplementary material for: Development and utility of SSR markers based on Brassica sp. whole-genome in triangle of U
Source: Front Plant Sci. 2024 Jan 8;14:1259736. doi: 10.3389/fpls.2023.1259736 (PMC10801002; doi:10.3389/fpls.2023.1259736)
Supplement: Supplementary Figure 1 — Transferability analysis on the designed SSR primers for the three basic species. (A), PCR amplification results of SSR primers for part of the AA genome; (B), PCR amplification results of SSR primers for part of the BB genome; C, PCR amplification results of SSR primers for part of the CC genome. [file DataSheet_1.zip › Supplementary Table 1.docx]

| **AA genome** |  |  |  |  |  |  |  |  |  |  |
| --- | --- | --- | --- | --- | --- | --- | --- | --- | --- | --- |
| **chromosomes** | A01 | A02 | A03 | A04 | A05 | A06 | A07 | A08 | A09 | A10 |
| 1. *rapa* (AA) | 7365 | 8190 | 9797 | 5754 | 7099 | 7263 | 7367 | 5905 | 11214 | 5172 |
| 1. *juncea* (AABB) | 7774  （5.55%↑） | 8403 （2.60%↑） | 9573 （2.29%↓） | 5451 （5.27%↓） | 6536 （7.93%↓） | 7154 （1.50%↓） | 6940 （5.80%↓） | 5656 （4.22%↓） | 11204 （0.09%↓） | 4893 （5.39%↓） |
| 1. *napus* (AACC) | 5007 （32.02%↓） | 5691 （30.51%↓） | 7141 （27.11%↓） | 4496 （21.86%↓） | 4970 （29.99%↓） | 5507 （24.18%↓） | 5657 （23.21%↓） | 4243 （28.15%↓） | 7844 （30.05%↓） | 3944 （23.74%↓） |
| **BB genome** |  |  |  |  |  |  |  |  |  |  |
| **chromosomes** | B01 | B02 | B03 | B04 | B05 | B06 | B07 | B08 |  |  |
| 1. *nigra* (BB) | 9988 | 13909 | 10547 | 10605 | 12720 | 10863 | 9875 | 12243 |  |  |
| 1. *juncea* (AABB) | 9215（7.74%↓） | 12452（10.48%↓） | 10853（2.90%↑） | 8546（19.42%↓） | 11047（13.15%↓） | 8444（22.27%↓） | 8265（16.30%↓） | 11327（7.48%↓） |  |  |
| 1. *carinata* (BBCC) | 14935（49.53%↑） | 12088（13.09%↓） | 11725（11.17%↑） | 10937（3.13%↑） | 10603（16.64%↓） | 10867（0.04%↑） | 10880（10.18%↑） | 8534（30.29%↓） |  |  |
| **CC genome** |  |  |  |  |  |  |  |  |  |  |
| **chromosomes** | C01 | C02 | C03 | C04 | C05 | C06 | C07 | C08 | C09 |  |
| 1. *oleracea* (CC) | 11883 | 14597 | 18286 | 15089 | 12966 | 11120 | 12806 | 12066 | 15250 |  |
| 1. *napus* (AACC) | 7315（38.44%↓） | 8209（43.76%↓） | 12561（31.31%↓） | 9525（36.87%↓） | 8504（34.41%↓） | 7399（33.46%↓） | 8733（31.81%↓） | 7780（35.52%↓） | 9058（40.60%↓） |  |
| 1. *carinata* (BBCC) | 16036（34.95%↑） | 13953（4.41%↓） | 13327（27.12%↓） | 13924（7.72%↓） | 13401（3.35%↑） | 13170（18.44%↑） | 12588（1.70%↓） | 11154（7.56%↓） | 10318（32.34%↓） |  |

**Table S1 Comparison of SSR loci number and distribution of subgenomic chromosomes between the three allotetraploids and three basic species**
