# Supplementary material for: Development and utility of SSR markers based on Brassica sp. whole-genome in triangle of U
Source: Front Plant Sci. 2024 Jan 8;14:1259736. doi: 10.3389/fpls.2023.1259736 (PMC10801002; doi:10.3389/fpls.2023.1259736)
Supplement: Supplementary Figure 1 — Transferability analysis on the designed SSR primers for the three basic species. (A), PCR amplification results of SSR primers for part of the AA genome; (B), PCR amplification results of SSR primers for part of the BB genome; C, PCR amplification results of SSR primers for part of the CC genome. [file DataSheet_1.zip › Supplementary Table 15.docx]

**Table S15 SSR primer information for cross-transferability in *B. nigra***

| **ID** | **Repeat** | **Sequence** | **Length** | **TM/℃** |
| --- | --- | --- | --- | --- |
| BniSSR00014 | AT | F:AGTGAAAACGCCTCTCTTTCC | 20 | 58.5 |
|  |  | R:AATATAAGAACATTAGCAGCAATGGG |  |  |
| BniSSR01556 | AAAC | F:CAGAGGCGCTGGTTAAAGC | 20 | 59.2 |
|  |  | R:TCTAGGGAAAGAGAAAAATCAAGAAGC |  |  |
| BniSSR01086 | AAAAG | F:CCAGGACTTTGAAGATGAACAAGC | 20 | 60.32 |
|  |  | R:ACTCGGCCGTTACAACTTCC |  |  |
| BniSSR06324 | A | F:CACTTGTCGCTTTCCATGGG | 12 | 59.48 |
|  |  | R:CAGCATTTACAAGCCGCTCC |  |  |
| BniSSR06730 | AAG | F:TCTCAACCCGTAACTTTTTATTTTTCC | 15 | 58.86 |
|  |  | R:GCAAATGGTTGAATTGAAAGAGAGG |  |  |
| BniSSR10277 | AAAAG | F:TCATGATGCTACCTCTAAGGACC | 25 | 59.1 |
|  |  | R:TTTGACCTTCCCCCTCATGC |  |  |
| BniSSR11896 | AAG | F:CCACCGGTTGCATCATAAAGC | 15 | 60.2 |
|  |  | R:TCGTGGCTTCTCTGTGTTGG |  |  |
| BniSSR15404 | AATCGT | F:AGTTAGTGTCTTCAGGGGAGC | 24 | 59.1 |
|  |  | R:GAGGTTTTCTCATGTGCCAGG |  |  |
| BniSSR15512 | A | F:TCACTTATGCAATTAGAAGTAACGGG | 12 | 59.46 |
|  |  | R:GATTAAACCACGCCTCCATGC |  |  |
| BniSSR15671 | ACTC | F:AGAAGCTCTGTTTTGGTAAATGATGG | 16 | 60.07 |
|  |  | R:GGCTTTGGGTTTGTCAAGGG |  |  |
|  |  |  |  |  |
| **ID** | **Repeat** | **Sequence** | **Length** | **TM/℃** |
| BniSSR17925 | A | F:CACCGTAGTTTTCTGTCCGC | 13 | 59.21 |
|  |  | R:GCCGCATTCTCTGGTTAAGC |  |  |
| BniSSR18916 | ACG | F:TTCAGGCTGTTTCTTTGGCG | 18 | 59.33 |
|  |  | R:ACTGGCGAAAAAGCACTTCG |  |  |
| BniSSR20265 | ATAGTG | F:TGGACAGATTTTCGAAGGATAGC | 24 | 58.56 |
|  |  | R:GTTCACCAATCTTGAGTTAATTAGCG |  |  |
| BniSSR23051 | AT | F:GTCATCACGAAAATAAAGGATTCAGC | 16 | 59.31 |
|  |  | R:ATTTGTCCAACTGCACCTCC |  |  |
| BniSSR23228 | AAAGG | F:GTGCAGCCCTAGATTGATGC | 20 | 59.05 |
|  |  | R:TTTCACATAATCACGTTGTCTTTACC |  |  |
| BniSSR25030 | ATC | F:TCTTCTGGCTGTTCGCTACC | 15 | 59.75 |
|  |  | R:GGAGGAGCATGGTATGATGAGC |  |  |
| BniSSR25056 | AAAAC | F:AGTTACATACGGTGTCTCAAAACG | 20 | 59.32 |
|  |  | R:ATATAAGTGGGTCATGTACATCTCG |  |  |
| BniSSR28810 | AAAAAG | F:CTTTGAAATTCAACCACTAAGGACC | 30 | 58.55 |
|  |  | R:TGATGTTTTTGACCTTCCCCC |  |  |
| BniSSR30437 | A | F:TGGTAAGTTATCTGGCCTCTGC | 12 | 59.83 |
|  |  | R:AGAAGCCAAACGAAAGATGCC |  |  |
| BniSSR29694 | AT | F:TGCTCCAAAATCATCACTTTACTCC | 70 | 59.58 |
|  |  | R:TTGACCCGTTTTACCCATGG |  |  |
| BniSSR34197 | AAAAG | F:GCAGATTCTCCTCCATCGTCG | 20 | 60.6 |
|  |  | R:CTTCCTCCGATAAGTCCTGCC |  |  |
| **ID** | **Repeat** | **Sequence** | **Length** | **TM/℃** |
| BniSSR34662 | ATAC | F:AGGAAAACAGAACAGAATCAGAAACC | 16 | 59.96 |
|  |  | R:AGCATGCGGGTTAAGAGACC |  |  |
| BniSSR34804 | A | F:TGCAAAAACCATGCTTTGTAATGC | 14 | 60.02 |
|  |  | R:TCGAGAGACGATGCTAGTGG |  |  |
| BniSSR36555 | AC | F:ATTAGACCATTTGATCACCTAGCC | 16 | 58.37 |
|  |  | R:ACAAGTTGTCTTAACCAGCCC |  |  |
| BniSSR38861 | AATGAG | F:CGTCTAGTGACTGAGTAAGAGCC | 42 | 59.94 |
|  |  | R:TCCTAGTCTAGACGCCCACC |  |  |
| BniSSR41502 | AGG | F:GCTGATCCGATACTCTTGGGG | 15 | 60 |
|  |  | R：AAGACGAAAACGCCGATTGC |  |  |
| BniSSR43298 | AAGGAG | F:TGATCCGTGGTGGTTCTTGG | 24 | 59.96 |
|  |  | R：CTATGTCCGGTGGTTCTCGG |  |  |
| BniSSR46366 | A | F:GGAAGAATACAAAGTGTCGGCG | 14 | 59.91 |
|  |  | R：AGCATTAAACCCACCCACCC |  |  |
| BniSSR45362 | AT | F:TGGACATTTTACTCCTGGAACG | 30 | 58.33 |
|  |  | R：TCAGGAACACTTCTAAATGTAACATCG |  |  |
| BniSSR44099 | AAAG | F:AAACAGATCTCGGTGGAGGC | 24 | 59.75 |
|  |  | R：AGTCCGGTTAAGCACAAGAGG |  |  |
| BniSSR48320 | C | F:AAAACCACTCCAACCCCACC | 12 | 60.4 |
|  |  | R：ACCACTAGACTGATCAGATTCAGC |  |  |
| BniSSR48412 | AAAAG | F:TCTCATGGTGCTACCTCTAAGG | 20 | 58.7 |
|  |  | R：TTTGACCTTCCCCCTCATGC |  |  |
|  |  |  |  |  |
| **ID** | **Repeat** | **Sequence** | **Length** | **TM/℃** |
| BniSSR49257 | AAG | F:ATCTTCCATTCCTCACCCGC | 15 | 59.82 |
|  |  | R：TGACGCTCAAATCCCACAGC |  |  |
| BniSSR52949 | AT | F:TCACTCTAAGATCAAGAGACGACC | 14 | 59.36 |
|  |  | R：GCTAGACAAATTTTGGAACGTTGG |  |  |
| BniSSR52978 | AAAC | F:ACTTTATTGTGGCCCTCCCC | 16 | 59.66 |
|  |  | R：AAGGTTTTCTAAATAAACACGACTGG |  |  |
| BniSSR58308 | AG | F:TGTAACATGTCACAATTAATCAAGGG | 18 | 58.17 |
|  |  | R：CAGAGAAGAACAGAGTGAAGAATCG |  |  |
| BniSSR57762 | AGGG | F:TGGGCGGAAGAACTTCTTGG | 16 | 60.25 |
|  |  | R：GAGAGACAGAGGGAGGGAGG |  |  |
| BniSSR57045 | A | F:CGAAGTGTGAGGATTTTGCTGG | 12 | 60.1 |
|  |  | R：ACAGTGGCAGCCAAAATAAGC |  |  |
| BniSSR61072 | AGCCGG | F:AGAAGTTTTCTCCGGAGCCG | 30 | 60.04 |
|  |  | R：CGGCTGAGAGAGACTATGGC |  |  |
| BniSSR60935 | AAACC | F:TGATTATCGAGGTCATGATCCTCC | 20 | 59.54 |
|  |  | R:TCAAGACTTGTAAACCTAACGTGC |  |  |

**Note:F denotes forward primer, R denotes reverse primer**
