# Supplementary material for: Development and utility of SSR markers based on Brassica sp. whole-genome in triangle of U
Source: Front Plant Sci. 2024 Jan 8;14:1259736. doi: 10.3389/fpls.2023.1259736 (PMC10801002; doi:10.3389/fpls.2023.1259736)
Supplement: Supplementary Figure 1 — Transferability analysis on the designed SSR primers for the three basic species. (A), PCR amplification results of SSR primers for part of the AA genome; (B), PCR amplification results of SSR primers for part of the BB genome; C, PCR amplification results of SSR primers for part of the CC genome. [file DataSheet_1.zip › Supplementary Table 16.docx]

**Table S16 SSR primer information for cross-transferability in *B. oleracea***

| **ID** | **Repeat** | **Sequence** | **Length** | **TM/℃** |
| --- | --- | --- | --- | --- |
| BolSSR000037 | AAG | F:GGGATCATCTTGTCCTCCCG | 18 | 59.86 |
|  |  | R:CTGGGCGGATGAAGAGACC |  |  |
| BolSSR000217 | ATGTGG | F:GTGCATTTTGTGTTTATATTTCAGAGC | 24 | 59.37 |
|  |  | R:TCATACATGCTTAGTGTCAACTGC |  |  |
| BolSSR002080 | AAAC | F:GCTTGCCTGATCATCATCGC | 16 | 60.07 |
|  |  | R:ACCTTATGTCCGAACCGAACC |  |  |
| BolSSR007683 | AG | F:CGCAGAGAAAGCAGAGAAGC | 24 | 60.11 |
|  |  | R:AAGTACCGACGCAAGAGACG |  |  |
| BolSSR016823 | A | F:TGTTCCTCGGAATTTCCTCGG | 17 | 59.84 |
|  |  | R:TCGTTCACGTAGAGGATGAACC |  |  |
| BolSSR011270 | AT | F:GTGGTGTCACAGTTCGGTCC | 42 | 60.01 |
|  |  | R:CCGTGCTGGCTTTTGAACC |  |  |
| BolSSR019203 | AACCG | F:TGAAACGACTAGGATCAAACCG | 20 | 59.2 |
|  |  | R:TCAAGTGGCATTTAACCGACG |  |  |
| BolSSR026022 | AAAG | F:ACGTCCTTGTCACTTAATTTCACC | 16 | 59.75 |
|  |  | R:TCCGACCGTAATCCAACACC |  |  |
| BolSSR028540 | AGG | F:CGAGGATGACGATCGTGAGG | 15 | 59.61 |
|  |  | R:CAACGTCTCTTGTATGCCACG |  |  |
| BolSSR025712 | C | F:ATCTAGCAAACCCTGGACGC | 20 | 60.32 |
|  |  | R:TGATGTCCAGCACGTCTTGG |  |  |
|  |  |  |  |  |
| **ID** | **Repeat** | **Sequence** | **Length** | **TM/℃** |
| BolSSR032727 | AAG | F:AAGAGACGCGGGTTTAGTGC | 18 | 60.54 |
|  |  | R:TGCTTCGTTGGAACTTCCCC |  |  |
| BolSSR029737 | A | F:CACGATCTTGAACCAACATCGG | 16 | 59.83 |
|  |  | R:CATCAAATCCCAACCACCGC |  |  |
| BolSSR036695 | AAGACC | F:TGAAATGGCAAAAACGTGAGC | 24 | 60.46 |
|  |  | R:GAGAGGAAGGCTCTTCACGC |  |  |
| BolSSR038500 | AAAT | F:GCTGAAGAGTTGGAGGTTGC | 20 | 60.39 |
|  |  | R:GTCACCCCTTGCTCTAACCG |  |  |
| BolSSR032608 | AAAAG | F:CAATAGATCACTGAATAGGTACGCC | 20 | 59.78 |
|  |  | R:TGGATTTCTTAAAAGCTGGTTCCG |  |  |
| BolSSR040411 | ACC | F:CATCTTGAAGGAGAAGCTGCC | 24 | 60.44 |
|  |  | R:CGTTGAATTCGATATAGCGTAACGG |  |  |
| BolSSR043303 | AAATAT | F:AAGAGTTCTGAGACAAGCTTGG | 24 | 59.4 |
|  |  | R:ACACAACTCTTAATGAAAATTCATGCC |  |  |
| BolSSR046422 | AG | F:AGGAGGGAAAGAACGATGACG | 16 | 59.82 |
|  |  | R:AACCCCACAAGTGACACTCC |  |  |
| BolSSR047956 | C | F:CGTCTTCTTCCTCCTCCTCG | 13 | 60.6 |
|  |  | R:TCACGTTAGCTTGCACAGGG |  |  |
| BolSSR045906 | AAAC | F:CCAACGAGGGGGAGATTAGG | 16 | 58.73 |
|  |  | R:AGTCTTAACTCTTCCCCACCC |  |  |
| BolSSR048557 | ATC | F:ATCCTCCTCCCCCATCATCC | 15 | 60.39 |
|  |  | R:ATCAAGCCGTAGCAAGGTGG |  |  |
|  |  |  |  |  |
| **ID** | **Repeat** | **Sequence** | **Length** | **TM/℃** |
| BolSSR048596 | AACCG | F:TATCCGAACCGAACCGAACC | 20 | 59.54 |
|  |  | R:TCGGATTTCGGGTAGTTCGG |  |  |
| BolSSR048778 | AAAC | F:AGCAAAGCTCAAGGAACTCC | 16 | 59.13 |
|  |  | R:GTTGTGTTGAGAGTTCAGACCC |  |  |
| BolSSR055158 | AAAACC | F:TCCAAGTTCTGAACTGCAAGC | 30 | 59.69 |
|  |  | R:TTGTGGCTTGATGTCGTTGC |  |  |
| BolSSR055223 | AT | F:CTCTTGTGAGATAATATGGGCACC | 26 | 60.01 |
|  |  | R:GTGCCTGCCTCTGTTTTGC |  |  |
| BolSSR055402 | AGG | F:AATGGGAATGGGAATGGGCG | 18 | 60.81 |
|  |  | R：CCGCCCATACGAGTTTGAGG |  |  |
| BolSSR057010 | AGTCG | F:GGGTGTCAAAATGAGCCAGC | 20 | 59.75 |
|  |  | R：TCGAGTTCGAGTTTACATATTGAAGC |  |  |
| BolSSR057359 | AG | F:AGTCTCTTCAAATTTGTTTGTCGG | 16 | 58.94 |
|  |  | R：CGGTTGAAATTTTTGATTTCGAAGC |  |  |
| BolSSR061403 | ATAG | F:CATATGTGGGCATGTGTACGC | 16 | 60.04 |
|  |  | R：GAACCAACACGAGGAGGAGG |  |  |
| BolSSR062978 | A | F:TGTGATGCTTTGTGACGTCG | 13 | 58.25 |
|  |  | R：TTTCGCGTCATTTTCTAATGGC |  |  |
| BolSSR065824 | AAG | F:TCACCTCTTCTTTTCTTCAGTGC | 39 | 59.5 |
|  |  | R：TCAAGCAGTGATTCCTCTACCC |  |  |
| BolSSR065902 | AACCG | F:TTCGGGTTTCGGGTAGTTCG | 20 | 59.83 |
|  |  | R：AACTATCCGAACCCGAACCG |  |  |
|  |  |  |  |  |
| **ID** | **Repeat** | **Sequence** | **Length** | **TM/℃** |
| BolSSR066145 | AAAAAT | F:ACTCTTTTTCATTCCCTTCCCC | 24 | 58.69 |
|  |  | R：TGCAACAAAGAACCTTCTCCC |  |  |
| BolSSR066317 | AT | F:TGATCTTCCTAATTGTCACGTTCG | 30 | 58.27 |
|  |  | R：ACTGAACTTGAAATGTTTAGGTTCG |  |  |
| BolSSR071914 | A | F:ACACTGATTCCATCTCTGTCTCC | 15 | 59.68 |
|  |  | R：TGTCGTTGCTAGGGTCAAGG |  |  |
| BolSSR071960 | ATAC | F:AGGCCCTCCGACTTGATAGG | 16 | 59.68 |
|  |  | R：TGCTCCCCCTTGTAAACTCG |  |  |
| BolSSR073495 | AGG | F:GACGATGATGAGCTCGAAGC | 24 | 59.18 |
|  |  | R：ACCGTCCCATACTCTTGAGC |  |  |
| BolSSR075430 | AAAAG | F:TTTTGGGGTGAAAGGTGTGG | 20 | 60.51 |
|  |  | R：CGCGTGCGCTTTTTCTACC |  |  |
| BolSSR081665 | A | F:GACGAAGAAGCTCCCATCCC | 13 | 59.11 |
|  |  | R：AGCTCCGTTAGGTCAGATATGG |  |  |
| BolSSR081414 | AG | F:TCTTCTCTCTCTCGGCTCGC | 18 | 59.39 |
|  |  | R:AGAACTGAGGAGGAGCAACG |  |  |

**Note:F denotes forward primer, R denotes reverse primer**
