# Supplementary material for: Development and utility of SSR markers based on Brassica sp. whole-genome in triangle of U
Source: Front Plant Sci. 2024 Jan 8;14:1259736. doi: 10.3389/fpls.2023.1259736 (PMC10801002; doi:10.3389/fpls.2023.1259736)
Supplement: Supplementary Figure 1 — Transferability analysis on the designed SSR primers for the three basic species. (A), PCR amplification results of SSR primers for part of the AA genome; (B), PCR amplification results of SSR primers for part of the BB genome; C, PCR amplification results of SSR primers for part of the CC genome. [file DataSheet_1.zip › Supplementary Table 14.docx]

**Table S14 SSR primer information for cross-transferability in *B. rapa***

| **ID** | **Repeat** | **Sequence** | **Length** | **TM/℃** |
| --- | --- | --- | --- | --- |
| BrSSR00004 | AT | F:GCTGCTTCTGCTCTTCATCC | 24 | 60.25 |
|  |  | R:ATGGTTGGTCCTTGGAGAGC |  |  |
| BrSSR01587 | ACC | F:AATCTCCGTCCAAAGCTCCG | 15 | 59.5 |
|  |  | R:CACTTCACATATCTCTCACGAAGC |  |  |
| BrSSR03830 | ACAG | F:ACACACATACACCTTCGAGACC | 20 | 60.0 |
|  |  | R:GACAAGAAGCCAGGAGACGG |  |  |
| BrSSR03677 | AAAAC | F:TGATGGATGAATGGATCACTTGG | 20 | 59.5 |
|  |  | R:CAAGGAGGAGCGACAATAAGC |  |  |
| BrSSR01972 | AAAAAG | F:TGTAGGGAGAGTGGCAAACG | 24 | 60.0 |
|  |  | R:ACTAGACAACAAAAATCCAATGTCG |  |  |
| BrSSR08077 | AG | F:TCAAAAGTAAACCATGTATAGGCCC | 14 | 59.9 |
|  |  | R:TTCTCGTCGCTTCACCTTCC |  |  |
| BrSSR06092 | AAATC | F:GTGCAGCCCTAGATTGATGC | 20 | 59.5 |
|  |  | R:TTTCACATAATCACGTTGTCTTTACC |  |  |
| BrSSR09075 | AAGCCC | F:AGAAGCTCTGTTTTGGTAAATGATGG | 24 | 59.6 |
|  |  | R:GGCTTTGGGTTTGTCAAGGG |  |  |
| BrSSR14973 | AT | F:TGGACAGATTTTCGAAGGATAGC | 24 | 60.1 |
|  |  | R:GTTCACCAATCTTGAGTTAATTAGCG |  |  |
| BrSSR15994 | AAAT | F:AAAACCACTCCAACCCCACC | 16 | 59.0 |
|  |  | R:ACCACTAGACTGATCAGATTCAGC |  |  |
|  |  |  |  |  |
|  |  |  |  |  |
| **ID** | **Repeat** | **Sequence** | **Length** | **TM/℃** |
| BrSSR13664 | AAAAT | F:TGGTAATATGCGTGCCCTCG | 20 | 60.2 |
|  |  | R:TCCTACATAAACTGGTCCCTACC |  |  |
| BrSSR11198 | AGAGGG | F:GATTCGCTTACTCGGCAAGC | 36 | 60.0 |
|  |  | R:GATTCGCTTACTCGGCAAGC |  |  |
| BrSSR17217 | A | F:AGCAAATGTCTTTGACCATCACG | 12 | 60.0 |
|  |  | R:TCTCCTCTGAACTCTCTGATGG |  |  |
| BrSSR18858 | AAG | F:CAGGGACTTGCCAAAATGCC | 21 | 59.5 |
|  |  | R:GCTGTAGCACAAGAGAGTTTTGG |  |  |
| BrSSR16468 | AGGGG | F:TCCGAGAGAGAGAGTGTGGG | 25 | 59 |
|  |  | R:GTATGGAGAACCTAAAATGAATCAAGG |  |  |
| BrSSR18217 | AACC | F:GAAGCAGAAGAGCCTCCTCC | 16 | 60 |
|  |  | R:GTTATGATTAGAGTGTTGAATCTCACC |  |  |
| BrSSR18436 | AACTC | F:AGTTTGGTCATAAAGCGGCG | 25 | 59.2 |
|  |  | R:AGAGCATGCAAAAGAGAAAGAGG |  |  |
| BrSSR21863 | A | F:ACCTATCCTTCAGTGCCACC | 12 | 29 |
|  |  | R:TGGGTTGATTCTAAGCCCACC |  |  |
| BrSSR23017 | AGAGGG | F:CGCTGGCTTTATCAATAACAGGG | 48 | 59.9 |
|  |  | R:CAACACATATCTTTCTCACCTTCCC |  |  |
| BrSSR24442 | ATAG | F:TGTAGGGAGAGTGGCAAACG | 16 | 59.5 |
|  |  | R:ACTAGACAACAAAAATCCAATGTCG |  |  |
| BrSSR25854 | AAG | F:ACCAGAGAGCTTGAGGAACG | 15 | 59.0 |
|  |  | R:TCCTCAGCCACTAGAGTTTCC |  |  |
|  |  |  |  |  |
| **ID** | **Repeat** | **Sequence** | **Length** | **TM/℃** |
| BrSSR28675 | AAAAG | F:TCTTCAAAAATAAGAAAGCGACAGC | 25 | 59.5 |
|  |  | R:GAATCGTTTGGGCATCACGG |  |  |
| BrSSR31751 | AATGGC | F:CACTGAGCAACTGTCAGAGC | 24 | 60 |
|  |  | R:TCGCCTTTTCCTTATTGCATCC |  |  |
| BrSSR32752 | AAAC | F:TCTCCATCCACCAGAGGAGG | 16 | 60 |
|  |  | R:CCATCTCACATGCATTACAAAACC |  |  |
| BrSSR33336 | ATATC | F:ACAGGTTTAGAATTGTATTCCCGC | 20 | 60.3 |
|  |  | R:CTCCAATTCCTAGAAAGCACGC |  |  |
| BrSSR34987 | AAAC | F:CCTTATTTGGTATACATGCTACTGCG | 16 | 59 |
|  |  | R：AGTGGTCAGGTCTGATGTTCC |  |  |
| BrSSR36313 | ACTGG | F:TCGATCTCAACCAAATTCAAGAGC | 20 | 59.5 |
|  |  | R：CCTCAAAGAAAACGAACTCCGC |  |  |
| BrSSR37152 | AT | F:AGAACATGCTTGTCTACTTCTTTTGG | 22 | 60.1 |
|  |  | R：CCAAAGGTGTACATTGTCCTTTCC |  |  |
| BrSSR37224 | A | F:CACTTGCACACCATCTGTTCG | 13 | 59.5 |
|  |  | R：TCAGACGACCCAAATTCCCC |  |  |
| BrSSR36383 | AAAG | F:ACACACATACACCTTCGAGACC | 16 | 59.3 |
|  |  | R：GACAAGAAGCCAGGAGACGG |  |  |
| BrSSR38719 | A | F:GTTTCAGGGGAAAATAAAAGTGAGC | 13 | 60.1 |
|  |  | R：CCATCTCCAACTCATTCATATATTTGC |  |  |
| BrSSR39889 | ATC | F:AGTAGTTGTAGCCGGTCAGG | 15 | 60.2 |
|  |  | R：TCAGTGTAAACCAGTGGCCC |  |  |
|  |  |  |  |  |
| **ID** | **Repeat** | **Sequence** | **Length** | **TM/℃** |
| BrSSR42556 | AAAAG | F:GGGGTCTGTCTAATTAAAACATCAGC | 20 | 58.5 |
|  |  | R：CGTTCGTGAGGCATTTGACG |  |  |
| BrSSR44040 | AG | F:AGATTCCATCCACAAAACAACCC | 14 | 60 |
|  |  | R：AGAACATAGTAGACGGAACTAGCG |  |  |
| BrSSR44329 | AAAAAC | F:CACGTTACACACCCGAATATGG | 30 | 59.4 |
|  |  | R：GGCACCAAAACTAAACAAACACC |  |  |
| BrSSR45708 | AACCG | F:GGGCTGTTCAATATGGTAAAACCG | 25 | 59.8 |
|  |  | R：CCAAACCATATCCAAATCCTACGG |  |  |
| BrSSR47369 | AGG | F:ACTTGGATCCCTGCTTGTCC | 18 | 59.5 |
|  |  | R：AAAGAAGATCTCGTCGCCGC |  |  |
| BrSSR48533 | AACT | F:ACTCGCTTTTCTACACAGTGC | 16 | 60.5 |
|  |  | R：AGATAGGCTGCATCCAAAGC |  |  |
| BrSSR48439 | C | F:ACTAAGTTTGAAAGCTCGACCC | 19 | 60.1 |
|  |  | R：GTGGTTACACATCAAGCATTTGG |  |  |
| BrSSR48597 | AC | F:CACCTTTTCCCACCCTTTGC | 22 | 60.0 |
|  |  | R：TGACAAAGCTCATCATCATTTACCC |  |  |

**Note:F denotes forward primer, R denotes reverse primer**
