# Supplementary material for: Development and utility of SSR markers based on Brassica sp. whole-genome in triangle of U
Source: Front Plant Sci. 2024 Jan 8;14:1259736. doi: 10.3389/fpls.2023.1259736 (PMC10801002; doi:10.3389/fpls.2023.1259736)
Supplement: Supplementary Figure 1 — Transferability analysis on the designed SSR primers for the three basic species. (A), PCR amplification results of SSR primers for part of the AA genome; (B), PCR amplification results of SSR primers for part of the BB genome; C, PCR amplification results of SSR primers for part of the CC genome. [file DataSheet_1.zip › Supplementary Table 17.docx]

**Table S17 Amplification results of the *B. nigra* cross-transferability test**

| **SSR** | ***B. rapa*** | ***B. nigra*** | ***B. oleracea*** | ***B. juncea*** | ***B. napus*** | ***B. carinata*** | ***A. thaliana*** | ***R. sativus*** |
| --- | --- | --- | --- | --- | --- | --- | --- | --- |
| BniSSR00014 | - | + | + | + | + | + | - | - |
| BniSSR01556 | - | + | - | + | - | + | + | - |
| BniSSR01086 | - | - | - | - | - | - | - | - |
| BniSSR06324 | - | + | - | + | - | + | + | + |
| BniSSR06730 | + | + | + | + | + | + | - | - |
| BniSSR10277 | - | + | - | + | - | - | - | - |
| BniSSR11896 | - | + | + | + | - | - | - | - |
| BniSSR15404 | + | + | + | + | + | + | + | - |
| BniSSR15512 | + | + | + | + | + | + | + | + |
| BniSSR15671 | + | + | + | + | + | + | + | - |
| BniSSR17925 | + | + | + | + | + | + | + | + |
| BniSSR18916 | + | + | + | + | + | + | - | - |
| BniSSR20265 | + | + | + | + | + | + | + | + |
| BniSSR23051 | + | + | + | + | + | + | - | - |
| BniSSR23228 | - | + | - | - | - | - | - | - |
| BniSSR25030 | - | + | - | + | - | + | + | - |
| BniSSR25056 | + | + | - | + | + | + | + | + |
| BniSSR28810 | - | - | - | - | - | - | - | - |
| BniSSR30437 | + | + | - | + | - | + | - | - |
| BniSSR29694 | + | + | + | - | + | + | - | - |
| BniSSR34197 | + | + | - | + | - | + | - | - |
| BniSSR34662 | - | + | - | + | - | + | + | - |
| Continued Table S17 |  |  |  |  |  |  |  |  |
| **SSR** | ***B. rapa*** | ***B. nigra*** | ***B. oleracea*** | ***B. juncea*** | ***B. napus*** | ***B. carinata*** | ***Arabidopsis thaliana*** | ***Raphanus sativus*** |
| BniSSR34804 | - | + | - | + | - | + | - | - |
| BniSSR36555 | - | + | - | - | - | - | + | - |
| BniSSR38861 | - | + | - | + | + | - | + | + |
| BniSSR41502 | + | + | + | + | + | + | + | - |
| BniSSR43298 | - | + | - | + | - | - | - | - |
| BniSSR46366 | - | + | - | + | - | + | + | + |
| BniSSR45362 | + | + | + | + | + | + | - | - |
| BniSSR44099 | + | + | + | + | + | + | + | + |
| BniSSR48320 | + | + | + | + | + | + | + | + |
| BniSSR48412 | + | + | + | + | - | + | - | - |
| BniSSR49257 | + | + | + | + | - | + | - | - |
| BniSSR52949 | - | + | + | + | + | + | + | - |
| BniSSR52978 | - | + | - | + | - | - | - | - |
| BniSSR58308 | + | + | + | + | + | + | + | - |
| BniSSR57762 | - | - | - | - | - | - | - | - |
| BniSSR57045 | + | + | + | + | + | + | + | - |
