# Supplementary material for: Development and utility of SSR markers based on Brassica sp. whole-genome in triangle of U
Source: Front Plant Sci. 2024 Jan 8;14:1259736. doi: 10.3389/fpls.2023.1259736 (PMC10801002; doi:10.3389/fpls.2023.1259736)
Supplement: Supplementary Figure 1 — Transferability analysis on the designed SSR primers for the three basic species. (A), PCR amplification results of SSR primers for part of the AA genome; (B), PCR amplification results of SSR primers for part of the BB genome; C, PCR amplification results of SSR primers for part of the CC genome. [file DataSheet_1.zip › Supplementary Table 18.docx]

**Table S18 Amplification results of the *B. oleracea* cross-transferability test**

| **SSR** | ***B. rapa*** | ***B. nigra*** | ***B. oleracea*** | ***B. juncea*** | ***B. napus*** | ***B. carinata*** | ***A. thaliana*** | ***R. sativus*** |
| --- | --- | --- | --- | --- | --- | --- | --- | --- |
| BolSSR000037 | + | + | + | + | + | + | - | + |
| BolSSR000217 | + | + | + | + | + | + | - | - |
| BolSSR002080 | + | + | + | + | + | + | - | + |
| BolSSR007683 | **-** | **-** | **+** | **-** | **+** | **+** | **-** | **-** |
| BolSSR016823 | + | + | + | + | + | + | + | + |
| BolSSR011270 | + | + | + | + | + | + | + | + |
| BolSSR019203 | - | - | + | + | + | + | - | - |
| BolSSR026022 | + | + | + | + | + | + | - | - |
| BolSSR028540 | **+** | **+** | **+** | **+** | **+** | **+** | **+** | **+** |
| BolSSR025712 | - | - | - | - | - | - | - | - |
| BolSSR032727 | + | + | + | + | + | + | + | - |
| BolSSR029737 | + | + | + | + | + | + | + | + |
| BolSSR036695 | + | + | + | + | + | + | + | + |
| BolSSR038500 | + | + | + | + | + | + | + | + |
| BolSSR032608 | + | + | + | + | + | + | + | + |
| BolSSR040411 | + | + | + | + | + | + | + | + |
| BolSSR043303 | **+** | **+** | **+** | **+** | **+** | **+** | **+** | - |
| BolSSR046422 | **+** | **+** | **+** | **+** | **+** | **+** | **+** | **+** |
| BolSSR047956 | - | - | + | - | - | + | - | - |
| BolSSR045906 | + | - | + | + | + | + | + | + |
| BolSSR048557 | + | + | + | + | + | + | + | + |
| Continued Table S18 |  |  |  |  |  |  |  |  |
| **SSR** | ***B. rapa*** | ***B. nigra*** | ***B. oleracea*** | ***B. juncea*** | ***B. napus*** | ***B. carinata*** | ***Arabidopsis thaliana*** | ***Raphanus sativus*** |
| BolSSR048596 | + | + | + | + | - | + | + | + |
| BolSSR048778 | + | + | + | + | + | + | - | - |
| BolSSR055158 | + | + | + | + | - | + | + | + |
| BolSSR055223 | **+** | **+** | **+** | **+** | **+** | **+** | **+** | **-** |
| BolSSR055402 | - | - | + | + | + | + | - | + |
| BolSSR057010 | + | - | + | + | - | - | + | - |
| BolSSR057359 | - | - | - | - | - | - | - | - |
| BolSSR061403 | - | + | + | + | + | + | + | + |
| BolSSR062978 | + | + | + | + | + | + | + | + |
| BolSSR065824 | - | - | + | + | + | + | - | - |
| BolSSR065902 | + | + | + | + | + | + | + | + |
| BolSSR066145 | + | + | + | + | + | + | + | - |
| BolSSR066317 | + | - | + | - | + | + | - | - |
| BolSSR071914 | + | + | + | + | + | + | + | + |
| BolSSR071960 | **+** | **-** | **+** | **+** | **+** | **+** | **+** | **+** |
| BolSSR073495 | + | + | + | - | - | + | + | - |
| BolSSR075430 | - | - | + | - | + | - | - | - |
