# Supplementary material for: Development and utility of SSR markers based on Brassica sp. whole-genome in triangle of U
Source: Front Plant Sci. 2024 Jan 8;14:1259736. doi: 10.3389/fpls.2023.1259736 (PMC10801002; doi:10.3389/fpls.2023.1259736)
Supplement: Supplementary Figure 1 — Transferability analysis on the designed SSR primers for the three basic species. (A), PCR amplification results of SSR primers for part of the AA genome; (B), PCR amplification results of SSR primers for part of the BB genome; C, PCR amplification results of SSR primers for part of the CC genome. [file DataSheet_1.zip › Supplementary Table 2.docx]

| **Table S2 Characteristics of SSR loci on each chromosome in *B. rapa*** | | | | | | | | | | |
| --- | --- | --- | --- | --- | --- | --- | --- | --- | --- | --- |
| Chromosome | A01 | A02 | A03 | A04 | A05 | A06 | A07 | A08 | A09 | A10 |
| Counts | 7365 | 8190 | 9797 | 5754 | 7099 | 7263 | 7367 | 5905 | 11214 | 5172 |
| GC content  (%) | 35 | 34.74 | 35.57 | 34.55 | 35.36 | 35.4 | 35.25 | 35.17 | 35.03 | 36.27 |
| Relative abundance (loci/Mb) | 251.11 | 262.01 | 261.45 | 263.16 | 250.09 | 250.74 | 255.3 | 258.09 | 250.32 | 250.04 |
