# Supplementary material for: Development and utility of SSR markers based on Brassica sp. whole-genome in triangle of U
Source: Front Plant Sci. 2024 Jan 8;14:1259736. doi: 10.3389/fpls.2023.1259736 (PMC10801002; doi:10.3389/fpls.2023.1259736)
Supplement: Supplementary Figure 1 — Transferability analysis on the designed SSR primers for the three basic species. (A), PCR amplification results of SSR primers for part of the AA genome; (B), PCR amplification results of SSR primers for part of the BB genome; C, PCR amplification results of SSR primers for part of the CC genome. [file DataSheet_1.zip › Supplementary Table 3.docx]

| **Table S3 Characteristics of SSR loci on each chromosome in *B. nigra*** | | | | | | | | |
| --- | --- | --- | --- | --- | --- | --- | --- | --- |
| Chromosome | B01 | B02 | B03 | B04 | B05 | B06 | B07 | B08 |
| Counts | 9988 | 13909 | 10547 | 10605 | 12720 | 10863 | 9875 | 12243 |
| GC content  (%) | 38.53 | 37.89 | 37.89 | 37.99 | 38.06 | 38.68 | 38.65 | 37.57 |
| Relative abundance (loci/Mb) | 173.94 | 196.32 | 191.5 | 184.64 | 186.04 | 178.62 | 169.99 | 181.86 |
