# Supplementary material for: Development and utility of SSR markers based on Brassica sp. whole-genome in triangle of U
Source: Front Plant Sci. 2024 Jan 8;14:1259736. doi: 10.3389/fpls.2023.1259736 (PMC10801002; doi:10.3389/fpls.2023.1259736)
Supplement: Supplementary Figure 1 — Transferability analysis on the designed SSR primers for the three basic species. (A), PCR amplification results of SSR primers for part of the AA genome; (B), PCR amplification results of SSR primers for part of the BB genome; C, PCR amplification results of SSR primers for part of the CC genome. [file DataSheet_1.zip › Supplementary Table 4.docx]

| **Table S4 Characteristics of SSR loci on each chromosome in *B. oleracea*** | | | | | | | | | |
| --- | --- | --- | --- | --- | --- | --- | --- | --- | --- |
| Chromosome | C01 | C02 | C03 | C04 | C05 | C06 | C07 | C08 | C09 |
| Counts | 11883 | 14597 | 18286 | 15089 | 12966 | 11120 | 12806 | 12066 | 15250 |
| GC content  (%) | 36.56 | 36.5 | 36.25 | 36.24 | 36.97 | 36.46 | 36.7 | 36.96 | 36.55 |
| Relative abundance (loci/Mb) | 226.51 | 221.17 | 245.43 | 230.64 | 224.03 | 232.44 | 228.85 | 231.76 | 226.11 |
