# Supplementary material for: Development and utility of SSR markers based on Brassica sp. whole-genome in triangle of U
Source: Front Plant Sci. 2024 Jan 8;14:1259736. doi: 10.3389/fpls.2023.1259736 (PMC10801002; doi:10.3389/fpls.2023.1259736)
Supplement: Supplementary Figure 1 — Transferability analysis on the designed SSR primers for the three basic species. (A), PCR amplification results of SSR primers for part of the AA genome; (B), PCR amplification results of SSR primers for part of the BB genome; C, PCR amplification results of SSR primers for part of the CC genome. [file DataSheet_1.zip › Supplementary Table 5.docx]

| **Table S5** **Characteristics of SSR loci on each chromosome in *B. juncea*** | | | | | | | | | | | | | | | | | | |
| --- | --- | --- | --- | --- | --- | --- | --- | --- | --- | --- | --- | --- | --- | --- | --- | --- | --- | --- |
| Chromosome | A01 | A02 | A03 | A04 | A05 | A06 | A07 | A08 | A09 | A10 | B01 | B02 | B03 | B04 | B05 | B06 | B07 | B08 |
| Counts | 7774 | 8403 | 9573 | 5451 | 6536 | 7154 | 6940 | 5656 | 11204 | 4893 | 9215 | 12452 | 10853 | 8546 | 11047 | 8444 | 8265 | 11327 |
| GC content(%) | 35.48 | 34.79 | 35.3 | 34.38 | 35.19 | 35.37 | 35.04 | 35.08 | 34.99 | 35.79 | 36.24 | 36.59 | 36.39 | 36.45 | 36.36 | 36.31 | 36.3 | 36.34 |
| Relative abundace(loci/Mb) | 228.14 | 246.54 | 253.17 | 245.11 | 234.61 | 243.43 | 245.08 | 241.36 | 236.81 | 257.56 | 243.67 | 246.74 | 245.7 | 235.56 | 231.46 | 265.28 | 235.36 | 221.51 |
