# Supplementary material for: Development and utility of SSR markers based on Brassica sp. whole-genome in triangle of U
Source: Front Plant Sci. 2024 Jan 8;14:1259736. doi: 10.3389/fpls.2023.1259736 (PMC10801002; doi:10.3389/fpls.2023.1259736)
Supplement: Supplementary Figure 1 — Transferability analysis on the designed SSR primers for the three basic species. (A), PCR amplification results of SSR primers for part of the AA genome; (B), PCR amplification results of SSR primers for part of the BB genome; C, PCR amplification results of SSR primers for part of the CC genome. [file DataSheet_1.zip › Supplementary Table 6.docx]

| **Table S6 Characteristics of SSR loci on each chromosome in *B. napus*** | | | | | | | | | | | | | | | | | | | |
| --- | --- | --- | --- | --- | --- | --- | --- | --- | --- | --- | --- | --- | --- | --- | --- | --- | --- | --- | --- |
| Chromosome | A01 | A02 | A03 | A04 | A05 | A06 | A07 | A08 | A09 | A10 | C01 | C02 | C03 | C04 | C05 | C06 | C07 | C08 | C09 |
| Counts | 5007 | 5691 | 7141 | 4496 | 4970 | 5507 | 5657 | 4243 | 7844 | 3944 | 7315 | 8209 | 12561 | 9525 | 8504 | 7399 | 8733 | 7780 | 9058 |
| GC content (%) | 35.12 | 34.54 | 35.23 | 34.46 | 35.22 | 35.53 | 34.74 | 35.13 | 34.65 | 36.09 | 36.72 | 36.43 | 36.05 | 36.17 | 36.92 | 36.24 | 36.35 | 36.74 | 36.64 |
| Relative abundance (loci/Mb) | 251.23 | 257.29 | 264.35 | 263.95 | 251.07 | 252.63 | 264.59 | 249 | 258.79 | 253.49 | 225.81 | 216.7 | 246.69 | 234.61 | 232.29 | 231.08 | 233.43 | 234.64 | 224.01 |
