# Supplementary material for: Development and utility of SSR markers based on Brassica sp. whole-genome in triangle of U
Source: Front Plant Sci. 2024 Jan 8;14:1259736. doi: 10.3389/fpls.2023.1259736 (PMC10801002; doi:10.3389/fpls.2023.1259736)
Supplement: Supplementary Figure 1 — Transferability analysis on the designed SSR primers for the three basic species. (A), PCR amplification results of SSR primers for part of the AA genome; (B), PCR amplification results of SSR primers for part of the BB genome; C, PCR amplification results of SSR primers for part of the CC genome. [file DataSheet_1.zip › Supplementary Table 7.docx]

| **Table S7 Characteristics of SSR loci on each chromosome in *B. carinata*** | | | | | | | | | | | | | | | | | |
| --- | --- | --- | --- | --- | --- | --- | --- | --- | --- | --- | --- | --- | --- | --- | --- | --- | --- |
| Chromosome | B01 | B02 | B03 | B04 | B05 | B06 | B07 | B08 | C01 | C02 | C03 | C04 | C05 | C06 | C07 | C08 | C09 |
| Counts | 14935 | 12088 | 11725 | 10937 | 10603 | 10867 | 10880 | 8534 | 16036 | 13953 | 13327 | 13924 | 13401 | 13170 | 12588 | 11154 | 10318 |
| GC content(%) | 37.72 | 38.55 | 38.32 | 38.48 | 38.77 | 38.19 | 38.03 | 39.05 | 36.01 | 36.14 | 36.38 | 36.31 | 36.54 | 36.35 | 36.32 | 36.27 | 36.58 |
| Relative abundace(loci/Mb) | 203.39 | 197.86 | 199.14 | 187.92 | 184.71 | 195.83 | 204.22 | 187.15 | 223.02 | 207 | 200.68 | 211.78 | 204.47 | 219.54 | 222.41 | 208.75 | 208.24 |
