# Supplementary figures and images for: Development and utility of SSR markers based on Brassica sp. whole-genome in triangle of U
Source: Front Plant Sci. 2024 Jan 8;14:1259736. doi: 10.3389/fpls.2023.1259736 (PMC10801002; doi:10.3389/fpls.2023.1259736)

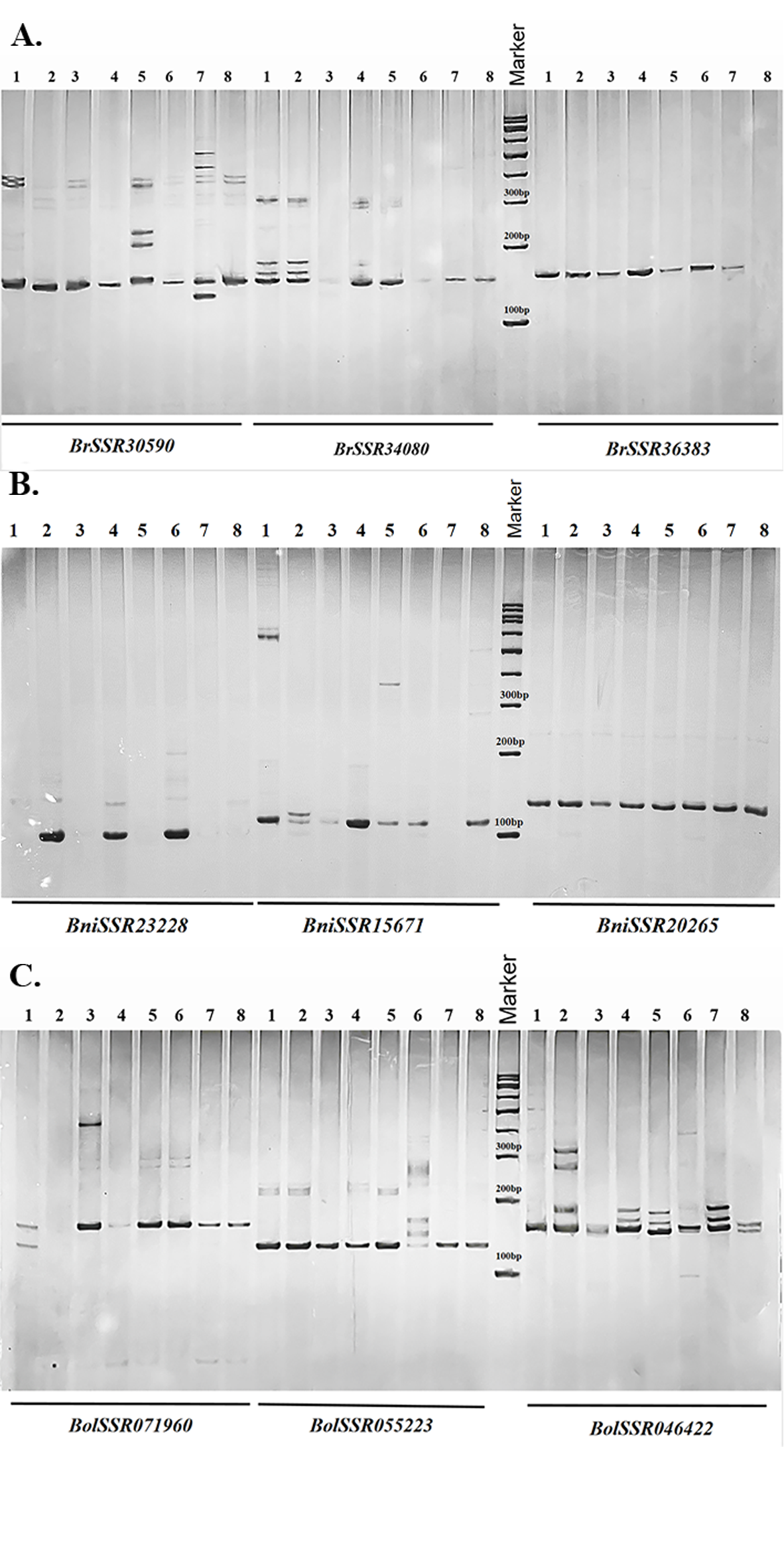

Supplement: Supplementary Figure 1 — Transferability analysis on the designed SSR primers for the three basic species. (A), PCR amplification results of SSR primers for part of the AA genome; (B), PCR amplification results of SSR primers for part of the BB genome; C, PCR amplification results of SSR primers for part of the CC genome. [file DataSheet_1.zip › Image 1 (55).TIF]

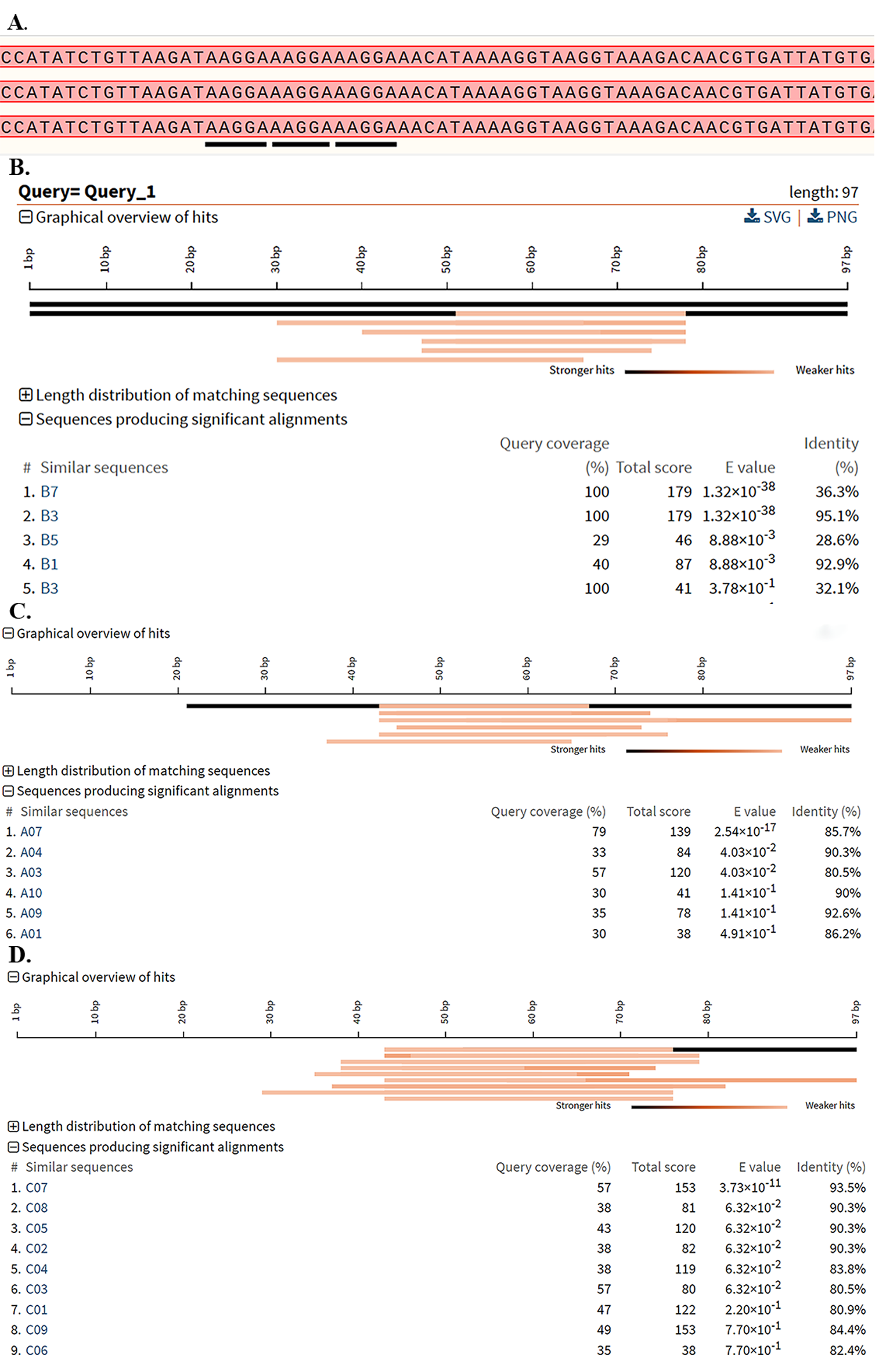

Supplement: Supplementary Figure 1 — Transferability analysis on the designed SSR primers for the three basic species. (A), PCR amplification results of SSR primers for part of the AA genome; (B), PCR amplification results of SSR primers for part of the BB genome; C, PCR amplification results of SSR primers for part of the CC genome. [file DataSheet_1.zip › Image 2 (32).TIF]
